# USP24-dependent STAT2 stabilization mediates physiologic and pathologic bone formation

**DOI:** 10.1038/s41419-026-08888-7

**Published:** 2026-05-30

**Authors:** Jung-Min Kim, Yeon-Suk Yang, Jun Xie, Chan Mi Kang, Yeong-Chan Cho, Kwang Hwan Park, Feng Gong, Tadatoshi Sato, Beom-Jik Kim, Guangping Gao, Jae-Hyuck Shim

**Affiliations:** 1 Department of Biology, College of Sciences, Kyung Hee University, Seoul, South Korea; 2 Department of Genetic and Cellular Medicine, Worcester, MA USA; 3 Department of Medicine, UMass Chan Medical School, Worcester, MA USA; 4 Horae Gene Therapy Center, UMass Chan Medical School, Worcester, MA USA; 5 Viral Vector Core, UMass Chan Medical School, Worcester, MA USA; 6 Department of Orthopaedic Surgery, Yonsei University College of Medicine, Seoul, South Korea; 7 Department of Biochemistry and Molecular Biology, University of Miami Miller School of Medicine, Miami, FL USA; 8 Li Weibo Institute for Rare Diseases Research, UMass Chan Medical School, Worcester, MA USA

**Keywords:** Bone development, Ubiquitylation, Cell signalling, Metabolic disorders

## Abstract

Osteoblast development must be precisely regulated, as insufficient bone formation results in low bone mass and skeletal fragility, whereas excessive osteogenesis drives heterotopic ossification (HO), the ectopic formation of bone in soft tissues. Here, we identify the deubiquitinating enzyme ubiquitin-specific peptidase 24 (USP24) as a key regulator of both physiological and pathological ossification. USP24 is highly expressed in skeletal tissues, where it promotes osteoblast development by stabilizing STAT2 through deubiquitination. Loss of USP24 reduces osteoblast differentiation and bone formation, an effect mirrored by STAT2 deficiency. Beyond physiologic bone, USP24 and STAT2 are also strongly expressed in heterotopic bones from patients with HO. In a fibrodysplasia ossificans progressiva (FOP) mouse model, recombinant adeno-associated virus (rAAV)-mediated silencing of *Usp24* markedly diminished HO pathogenesis and reduced STAT2 protein levels in HO lesions. Consistently, USP24 deficiency attenuated activin A-induced bone morphogenetic protein (BMP) signaling and osteogenesis, with comparable effects observed upon *Stat2* silencing. Together, these findings uncover a previously unrecognized role for USP24-STAT2 signaling in osteoblast differentiation and HO, highlighting bone-targeted USP24-STAT2 inhibition as a potential therapeutic strategy for pathologic bone formation.

## Introduction

Bone is a dynamic tissue that undergoes continuous remodeling, a process tightly regulated by the balance between bone-resorbing osteoclasts and bone-forming osteoblasts [1, 2]. Osteoblasts arise from mesenchymal stem cells, which commit to the osteoprogenitor lineage, progress into pre-osteoblasts, proliferate, and then differentiate into mature osteoblasts that produce extracellular matrix proteins and mineral. Mature osteoblasts terminally differentiate into osteocytes, embedding themselves within the bone matrix [3]. These developmental processes must be precisely regulated in a tissue- and context-dependent manner, as insufficient bone formation leads to osteoporosis, while excessive bone formation results in ectopic ossification.

Heterotopic ossification (HO)—the abnormal formation of bone within soft tissues—can occur sporadically following burns, traumatic brain injury, fractures, dislocations, or surgical procedures, often resulting in restricted joint mobility, severe pain, and nerve entrapment [4–7]. HO is postulated to reflect the aberrant differentiation of muscle-resident fibroadipogenic progenitors into osteoblasts [8]. The most severe form of HO is fibrodysplasia ossificans progressiva (FOP, #OMIM 135100), an ultra-rare genetic disorder characterized by disabling HO within skeletal muscle, tendons, ligaments, fascia, and aponeuroses [9–11]. In FOP, HO typically begins in childhood or early adulthood and progresses through episodic, painful inflammatory swellings (“flare-ups”) that occur spontaneously or are triggered by minor trauma, intramuscular injection, or local inflammation. Over time, cumulative HO leads to profound immobility and chronic, severe pain [12].

This study identifies the deubiquitinating enzyme ubiquitin-specific peptidase 24 (USP24) as a mediator of both physiological and pathological bone formation through its regulation of osteoblast differentiation. USP24, a member of the ubiquitin-specific peptidase (USP) family, functions as a deubiquitinating enzyme (DUB) that modulates diverse cellular processes by reversing polyubiquitination or monoubiquitination of target proteins [13]. Our previous study has identified USP24 as a putative interacting partner of RUNX2, a master regulator of osteoblast development, using unbiased mass spectrometry analysis. USP24 deficiency impairs osteoblast differentiation but is dispensable for RUNX2 stabilization [14]. To date, most studies of USP24 have focused on cancer, where it has been implicated in tumorigenesis, upregulation in tumor-associated macrophages, and regulation of cell cycle, apoptosis, and mitotic fidelity [15–17]. Genetic deletion of *Usp24* results in neonatal lethality, with pups born alive but a subset dying within h of birth for unknown reasons [18]. However, the roles of USP24 in skeletal biology remain largely unexplored.

Here, we demonstrate that USP24 promotes osteoblast differentiation and bone formation by stabilizing signal transducer and activator of transcription 2 (STAT2). The STAT family comprises STAT1, STAT2, STAT3, STAT4, STAT5 (STAT5A and STAT5B), and STAT6 [19]. Among these, only STAT3 has been previously shown to act as a bone-forming activator [20, 21]. However, how posttranslational mechanisms regulate STAT2 during osteoblast lineage commitment and maintain its activity to drive differentiation remains unclear. Our findings establish that the USP24-STAT2 signaling axis functions as a critical regulator of osteoblast development, fine-tuning both normal and aberrant bone formation.

## Results

### USP24 is required for osteoblast development

**Fig. 1:**
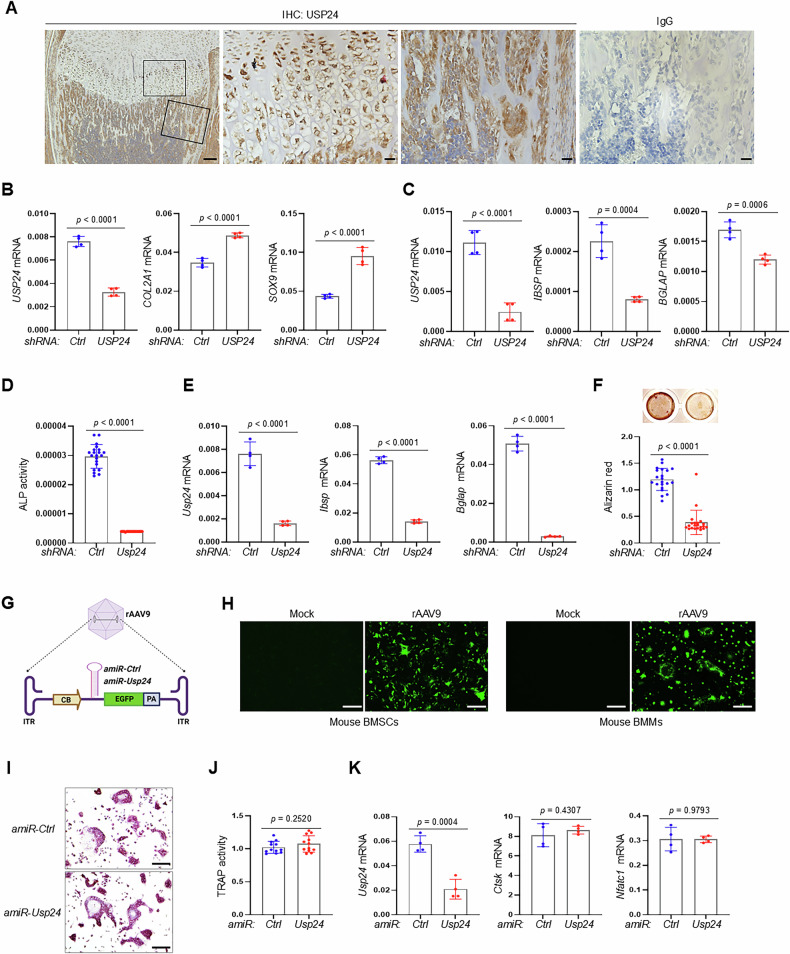
USP24 is required for osteogenic differentiation. **A** Immunohistochemistry (IHC) for USP24 in the mouse femur at postnatal day 10. USP24 expression was detected in osteoblasts of the trabecular bone and chondrocytes of the growth plate. IgG was used for negative staining. Scale bars, 100 μm (left); 20 μm (right, enlarged view). Human bone marrow-derived mesenchymal stem cells (BMSCs) expressing control shRNA (*shCtrl*) or *USP24* shRNA (*shUSP24*) were cultured under chondrogenic or osteogenic conditions. Expression of chondrocyte-specific genes (**B**) and osteoblast-specific genes (**C**) was assessed by qRT-PCR at day 14 and day 12, respectively (*n* = 4). Mouse calvarial osteoblast (COB) cells infected with lentiviruses expressing *shCtrl* (control) or *shUsp24* (*Usp24* knockdown) were cultured under osteogenic conditions. Alkaline phosphatase (ALP) activity (**D**, *n* = 21) and osteoblast gene expression (**E**, *n* = 4) were analyzed on day 6 of osteogenic culture. (**F**) Mineralization was determined by alizarin red staining at day 30 of osteogenic culture (*n* = 21). **G** Schematic diagram showing the rAAV9 construct that contains a CMV enhancer/chicken β-actin promoter (CB), *amiR-Ctrl*, *amiR-Usp24*, an *Egfp* reporter gene (enhanced green fluorescent protein, EGFP), *β-globin* polyA sequence (PA), and inverted terminal repeat (ITR). **H** Phosphate-buffered saline (PBS, mock) or 5 × 10^10^ genome copies (GCs) of rAAV9.egfp were treated to mouse BMSCs or mouse bone marrow monocytes (BMMs), and EGFP expression was assessed by fluorescence microscopy at 3 day post-infection. Scale bar, 200 μm. 5 ×10^10^ GCs of rAAV9.egfp carrying *amiR-Ctrl* or *amiR-Usp24* were introduced to mouse BMMs, and cells were cultured under osteoclast differentiation conditions for 6 days. Osteoclast differentiation was assessed by tartrate-resistant acid phosphatase (TRAP) staining (**I**), TRAP activity (**J**, *n* = 12), and osteoclast gene expression (**K**, *n* = 4). Scale bar, 125 μm (**I**). Data are representative of three (**A–F, H–K**). A two-tailed unpaired Student’s *t* test for comparing two groups (**B-F, J, K**; error bars, SD of biological replicates).

To investigate the role of USP24 in the skeleton, we first examined its expression during skeletal development (Fig. 1A). Immunohistochemistry (IHC) revealed USP24 expression in growth plate chondrocytes and trabecular osteoblasts at postnatal day 10 (P10). To assess its functional significance, human bone marrow–derived mesenchymal stromal cells (BMSCs) were transduced with lentiviruses expressing either a control shRNA (*shCtrl*) or a USP24-targeting shRNA (*shUSP24*) and cultured under either chondrogenic or osteogenic differentiation conditions. *USP24* silencing enhanced chondrogenic differentiation, as evidenced by increased expression of chondrogenic markers including collagen type II alpha 1 (*COL2A1*) and SRY-box transcription factor 9 (*SOX9*) (Fig. 1B). Conversely, *USP24*-deficient BMSCs displayed impaired osteoblast differentiation, with reduced expression of osteoblast markers such as bone sialoprotein 2 (*IBSP*) and osteocalcin (*BGLAP*) (Fig. 1C). Together, these findings suggest that USP24 promotes osteoblast lineage commitment while restraining the chondrogenic potential of skeletal progenitors.

Next, to examine USP24 function after stem cell commitment to the osteoblast lineage, mouse calvarial osteoblasts (COBs) were transduced with lentiviruses expressing either *shCtrl* or *shUSP24*. *Usp24* knockdown markedly reduced alkaline phosphatase (ALP) activity, expression of osteoblast-specific genes, such as *Ibsp* and *Bglap*, and matrix mineralization as assessed by Alizarin Red staining (Fig. 1D–F). To further confirm the role of USP24 in osteoblast differentiation and bone formation, we employed a bone-targeting recombinant adeno-associated virus (rAAV) vector carrying an artificial miRNA against *Usp24* (*amiR-Usp24*) that silences *Usp24* expression (Fig. 1G). Consistent with previous reports establishing rAAV9 as an efficient vector for osteoblast- and osteoclast-lineage transduction in vitro and in vivo²¹, we validated robust rAAV9-mediated enhanced green fluorescent protein (EGFP) expression in mouse BMSCs-derived osteoblasts and bone marrow monocytes (BMMs)-derived osteoclasts (Fig. 1H). We then tested the effect of *Usp24* silencing on lineage-specific differentiation. In osteoblast cultures, *amiR-Usp24* significantly reduced ALP staining (Supplementary Fig. S1A), ALP activity (Supplementary Fig. S1B), and expression of osteoblast markers, including collagen type I alpha 1 (*Col1a1*) and *Bglap* (Supplementary Fig. S1C). By contrast, in osteoclast cultures, *amiR-Usp24* had no detectable effect on tartrate-resistant acid phosphatase (TRAP) staining (Fig. 1I), TRAP activity (Fig. 1J), or expression of osteoclast-specific genes such as cathepsin K (*Ctsk*) and nuclear factor of activated T cells 1 (*Nfatc1*) (Fig. 1K). These findings indicate that USP24 is essential for osteoblast differentiation but dispensable for osteoclast development.

### USP24 promotes bone formation in vivo

**Fig. 2:**
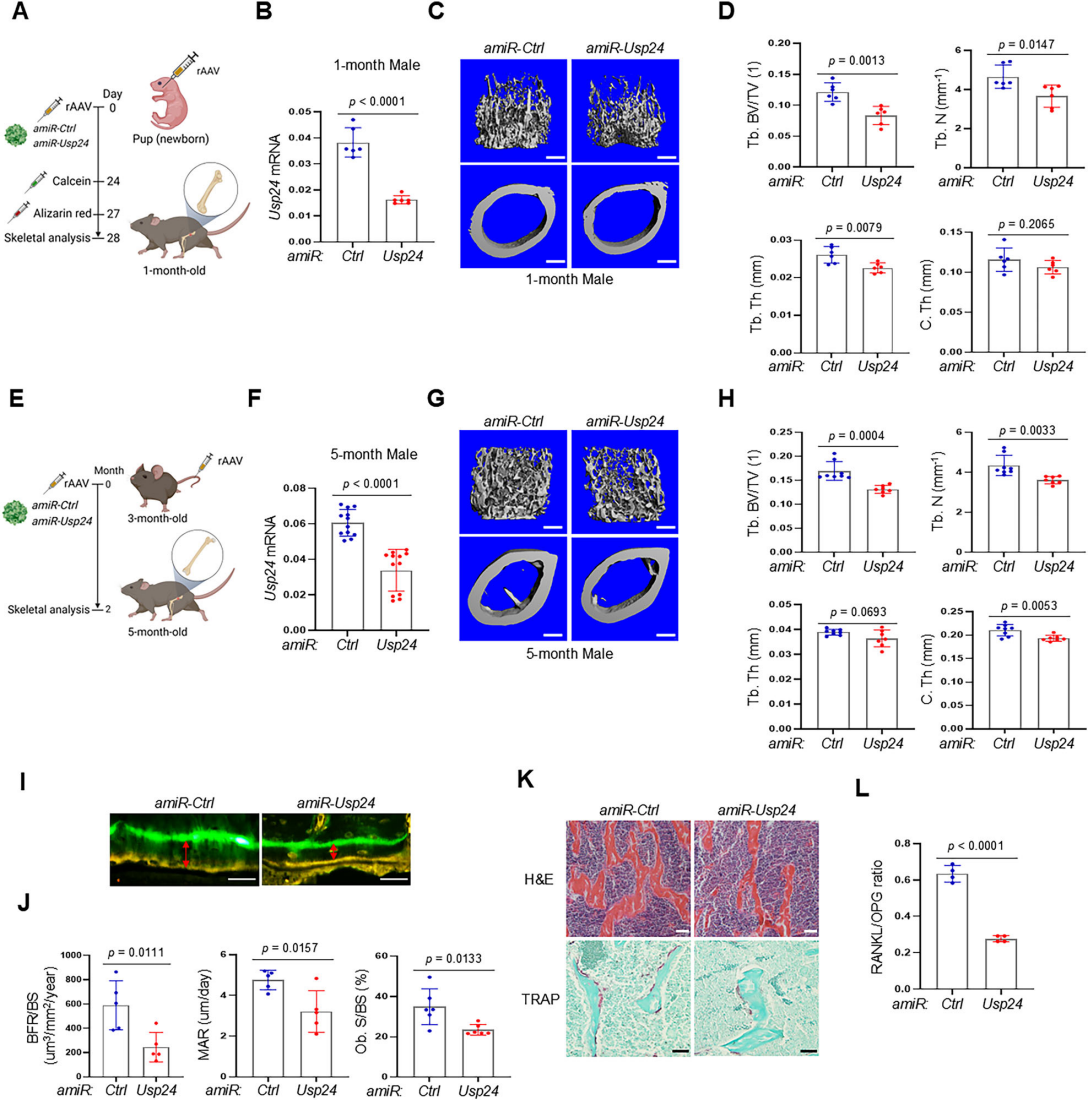
USP24 is required for in vivo bone formation and skeletal homeostasis. **A** Schematic diagram showing the experimental strategy for rAAV injection into neonates at postnatal day 0 (P0). 10^11^ GCs of rAAV9.egfp carrying *amiR-Ctrl* or *amiR-Usp24* were injected into P0 neonates and 28 days later, *Usp24* mRNA levels in rAAV-treated tibia were assessed by qRT-PCR (**B**, *n* = 6). Micro-computed tomography (Micro-CT) analysis showing trabecular bone mass and cortical bone thickness in rAAV-treated femurs. Representative 3D-reconstruction (**C**) and relative quantification (**D**, *n* = 6) are displayed. Trabecular bone volume/total volume (Tb. BV/TV), trabecular number (Tb. N), trabecular thickness (Tb. Th), and cortical thickness (C. Th) were measured. Scale bar, 500 μm (**C**). **E** Schematic diagram showing the experimental strategy for rAAV injection into 3-month-old male mice. (F-H) 5 × 10^13^ vector genome (vg)/kg of rAAV9.egfp carrying *amiR-Ctrl* or *amiR-Usp24* was injected into 3-month-old male mice, and 2 months later, *Usp24* mRNA levels in rAAV-treated tibia were assessed by qRT-PCR (**F**, *n* = 12). Micro-CT analysis showing trabecular bone mass and cortical bone thickness in rAAV-treated femurs. Representative 3D-reconstruction (**G**) and relative quantification (**H**, *n* = 8 for *amiR-Ctrl* and *n* = 7 for *amiR-Usp24*) are displayed. Scale bar, 500 μm (**G**). Dynamic histomorphometric analysis of 1-month-old rAAV-treated femurs (corresponding to panel 2a). Representative images of calcein/alizarin red labeling in cortical bone (**I**) and relative histomorphometric quantification of BFR/BS, MAR, and Ob. S/BS (**J**, *n* = 5). Scale bar, 50 μm (**I**). BFR/BS, bone formation rate per bone surface; MAR, mineral apposition rate; Ob. S/BS, osteoblast surface per bone surface. **K** Hematoxylin and eosin (H&E)-stained (top) and TRAP-stained (bottom) longitudinal sections of 1-month-old rAAV-treated femurs. Scale bar, 50 μm. **L** mRNA levels of a RANKL/OPG ratio in rAAV-treated tibia were assessed by qRT-PCR (*n* = 4). Data are representative of three (**C, G, I, K, L**). A two-tailed unpaired Student’s t-test for comparing two groups (**B, D, F, H, J, L**; error bars, SD of biological replicates).

To explore the role of USP24 in early skeletal development, rAAV9 vectors carrying either *amiR-Ctrl* or *amiR-Usp24* were injected intravenously (i.v.) into wild-type neonates at postnatal day 0 (P0). Bone phenotypes were assessed 4 weeks later by micro-computed tomography (micro-CT) and histological analysis (Fig. 2A–D and Supplementary Fig. S2). *amiR-Usp24* treatment reduced *Usp24* mRNA levels in the tibia by ~50–60%, as confirmed by quantitative RT-PCR (qRT-PCR) (Fig. 2B). Micro-CT analysis revealed that compared with *amiR-Ctrl*, *amiR-Usp24* markedly decreased trabecular bone mass, as evidenced by reductions in bone volume per tissue volume (Tb. BV/TV), trabecular number (Tb. N), and trabecular thickness (Tb. Th), whereas cortical bone parameters were largely unaffected (Fig. 2C, D). A similar phenotype was observed in female mice, with decreased tibial *Usp24* expression (Supplementary Fig. S2A) accompanied by significant reductions in both trabecular and cortical bone mass (Supplementary Fig. S2B, C). Importantly, histological evaluation of non-skeletal tissues, including the kidney, spleen, liver, lung, and heart, showed no apparent abnormalities following *amiR-Usp24* treatment (Supplementary Fig. S2D). We next examined the role of USP24 in maintaining bone homeostasis in adult mice. 3-month-old mice were injected i.v. With rAAV9 vectors carrying either *amiR-Ctrl* or *amiR-Usp24*, skeletal phenotypes were analyzed 2 months later (Fig. 2E). Robust knockdown of *Usp24* in tibial bones was confirmed by qRT-PCR (Fig. 2F). Consistent with earlier observations, *amiR-Usp24*-treated mice exhibited significant reductions in both trabecular and cortical bone, as determined by micro-CT analysis (Fig. 2G, H).

Dynamic histomorphometry further revealed that *amiR-Usp24* treatment reduced mineral apposition rate (MAR), bone formation rate (BFR), and osteoblast surface relative to bone surface (Ob.S/BS) in the femur (Fig. 2I, J). Histological analysis also showed a substantial decrease in TRAP-positive osteoclasts on bone surfaces in *amiR-Usp24*-treated mice (Fig. 2K). This reduction in osteoclast numbers was accompanied by a decreased receptor activator of nuclear factor kappa-Β ligand (RANKL)/osteoprotegerin (OPG) ratio in tibial bone (Fig. 2L), indicating that *Usp24* silencing impacts not only bone formation but also bone resorption. A similar reduction in osteoclast numbers and RANKL/OPG ratio was observed in 5-month-old *amiR-Usp24*-treated mice (Supplementary Fig. S3). Notably, this decrease in the RANKL/OPG ratio was also observed in *Usp24*-deficient osteoblasts (Supplementary Fig. S1D). Together with our findings that USP24 deficiency does not affect osteoclast development (Fig. 1I–K), these results suggest that USP24 indirectly regulates osteoclast development through modulation of the osteoblast-derived RANKL/OPG balance. Collectively, these findings suggest that USP24 deficiency leads to low bone mass by suppressing osteoblast-driven bone formation and altering overall bone remodeling.

### USP24 regulates STAT2 protein stability in osteoblasts

**Fig. 3:**
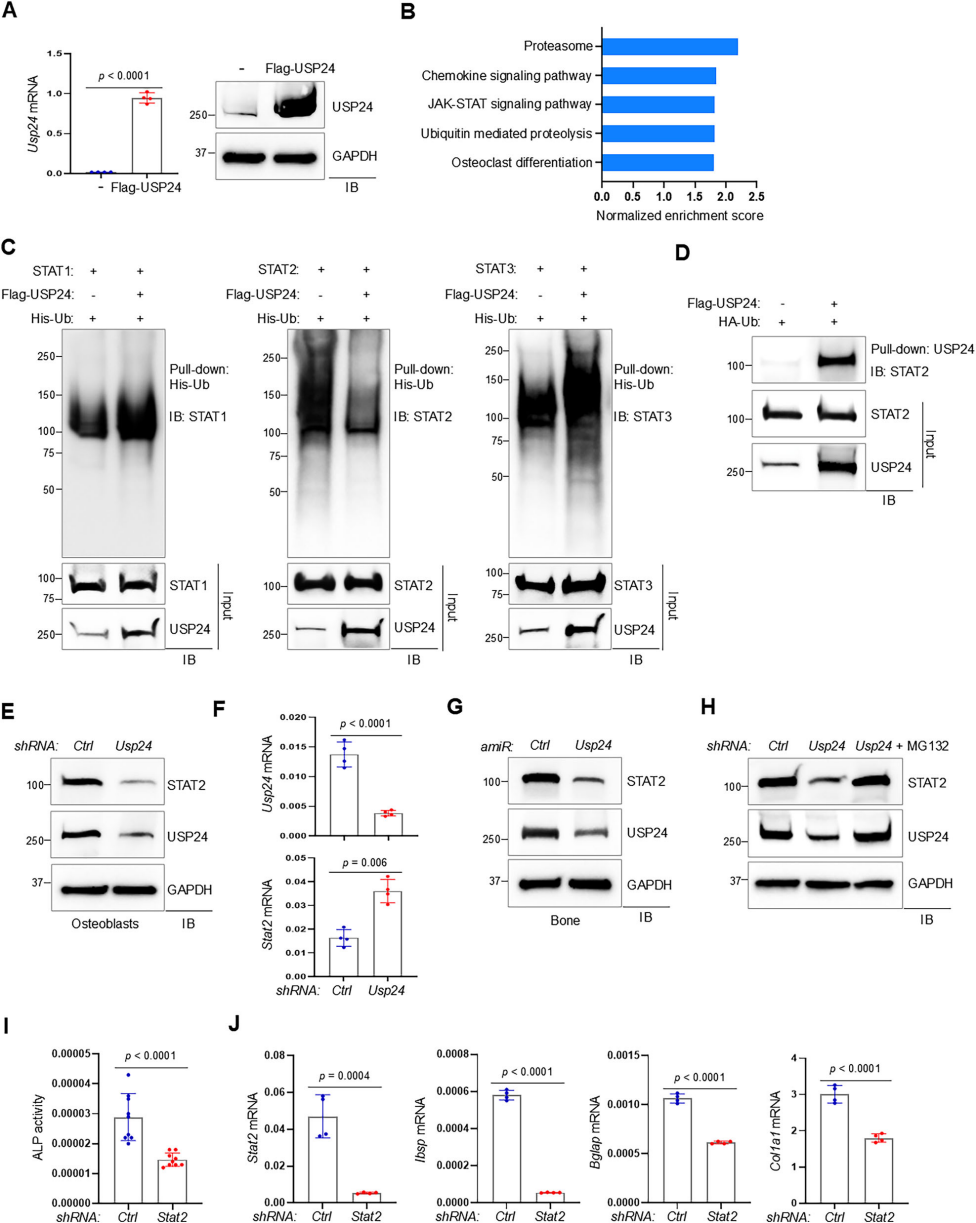
USP24 controls STAT2 protein stability in osteoblasts. **A** C3H10T1/2 cells were transfected with either vector control or Flag-*USP24*, and 48 h later, mRNA levels (left, *n* = 4) and protein levels (right) of USP24 were determined by qRT-PCR and immunoblotting, respectively. **B** Enrichment scores of putative USP24-binding proteins generated by WebGestalt pathway analysis. **C** Flag-*USP24* and His-ubiquitin were transfected into HEK293T cells along with *STAT1*, *STAT2*, or *STAT3*. After 48 h of transfection, cells were treated with 10 μM MG132 for 6 h, lysed, pulled down with Ni-NTA agarose, and immunoblotted with the indicated antibodies. **D** Flag-*USP24* and HA-ubiquitin were transfected into C3H10T1/2 cells. 48 h later, cells were treated with 10 μM MG132 for 6 h, lysed, pulled down with Flag-conjugated agarose, and immunoblotted for STAT2. STAT2 protein levels (**E**) and mRNA levels (**F**, *n* = 4) in *shCtrl*- or *shUsp24*-expressing mouse COBs. **G** 10^11^ GCs of rAAV9.egfp carrying *amiR-Ctrl* or *amiR-Usp24* were injected into P0 neonates, and 28 days later, STAT2 protein levels in the tibia were assessed by immunoblotting. **H** *shCtrl* or *shUsp24*-expressing mouse COBs were treated with 10 μM MG132 for 6 h and immunoblotted for STAT2. *shCtrl* or *shStat2*-expressing mouse COBs were cultured under osteogenic conditions. Six days later, ALP activity (**I**, *n* = 9) and osteoblast gene expression (**J**, *n* = 4) were examined. Data are representative of three (**A, C–J**). A two-tailed unpaired Student’s *t* test for comparing two groups (**A, F, I, J**; error bars, SD of biological replicates).

To identify potential USP24-binding partners in osteoblasts, affinity purification using Flag-tagged USP24 protein followed by mass spectrometry was performed using C3H10T1/2 cells, a mouse fibroblast-like mesenchymal stem cell line (Fig. 3A). Pathway analysis of USP24-binding proteins revealed enrichment in several signaling pathways, including the proteasome and Janus kinase (JAK)-Signal transducer and activator of transcription (STAT) signaling pathway (Fig. 3B). Within the JAK-STAT signaling pathway, STAT1 and STAT2 were specifically enriched, suggesting that they may serve as potential USP24-binding partners.

In vitro deubiquitination assays showed that USP24 overexpression induced STAT2 deubiquitination, whereas ubiquitination levels of STAT1 and STAT3 remained unaffected by USP24 overexpression (Fig. 3C). Co-immunoprecipitation analysis further confirmed a physical interaction between USP24 and STAT2 in osteoblasts (Fig. 3D). Lentiviral shRNA-mediated knockdown of *Usp24* led to a substantial reduction in STAT2 protein levels in osteoblasts, while *Stat2* mRNA levels were upregulated, likely as a compensatory response (Fig. 3E, F). Consistently, tibial bones from *amiR-Usp24*-treated mice also exhibited decreased STAT2 protein levels (Fig. 3G). However, treatment of the proteasome inhibitor MG132 restored STAT2 levels in *Usp24*-deficient osteoblasts (Fig. 3H), indicating that USP24-mediated deubiquitination stabilizes STAT2 in osteoblasts by preventing its ubiquitin-dependent proteasomal degradation. Accompanied by reduced protein levels of STAT2, its phosphorylation levels were decreased in *Usp24*-deficient osteoblasts following stimulation with interferon (IFN)-α (Supplementary Fig. S4A). STAT2 mediates activation of IFN-stimulated response elements (ISREs) by forming complexes with other STAT proteins or IFN regulatory factor 9 (IRF9), which in turn leads to transcription of IFN-stimulated genes (ISGs) [22, 23]. *Usp24*-deficient osteoblasts also showed a significant decrease in ISRE-luc reporter activity (Supplementary Fig. S4B) and ISGs expression (Supplementary Fig. S4C), indicating that USP24 deficiency decreases both protein and phosphorylation levels of STAT2 in osteoblasts, resulting in diminished STAT2 transcription activity.

Given that the role of STAT2 in osteoblasts is largely uncharacterized, we examined the effect of *Stat2* knockdown in mouse pre-osteoblasts. As seen in *Usp24*-deficient osteoblasts, STAT2 deficiency led to reduced ALP activity (Fig. 3I) and decreased expression of osteoblast-specific genes, including *Ibsp, Bglap*, *Col1a1*, runt-related transcription factor 2 (*Runx2*), and osterix (*Sp7*) (Fig. 3J and Supplementary Fig. S5A). In addition, the RANKL/OPG ratio was decreased in these cells (Supplementary Fig. S5B), mirroring the phenotype observed in *Usp24*-deficient osteoblasts. Notably, TRAP activity, formation of multinucleated osteoclasts, and osteoclast gene expression were all normal in *Stat2*-deficient osteoclasts (Supplementary Fig. S6), demonstrating that STAT2 is dispensable for osteoclast development. These results suggest that USP24 and STAT2 function within the same signaling axis to control osteoblast and osteoclast biology. The USP24-STAT2 signaling axis is critical for osteoblast differentiation, while it is likely to regulate osteoclast development indirectly by modulating the osteoblast-derived RANKL/OPG balance.

### USP24 mediates heterotopic ossification

**Fig. 4:**
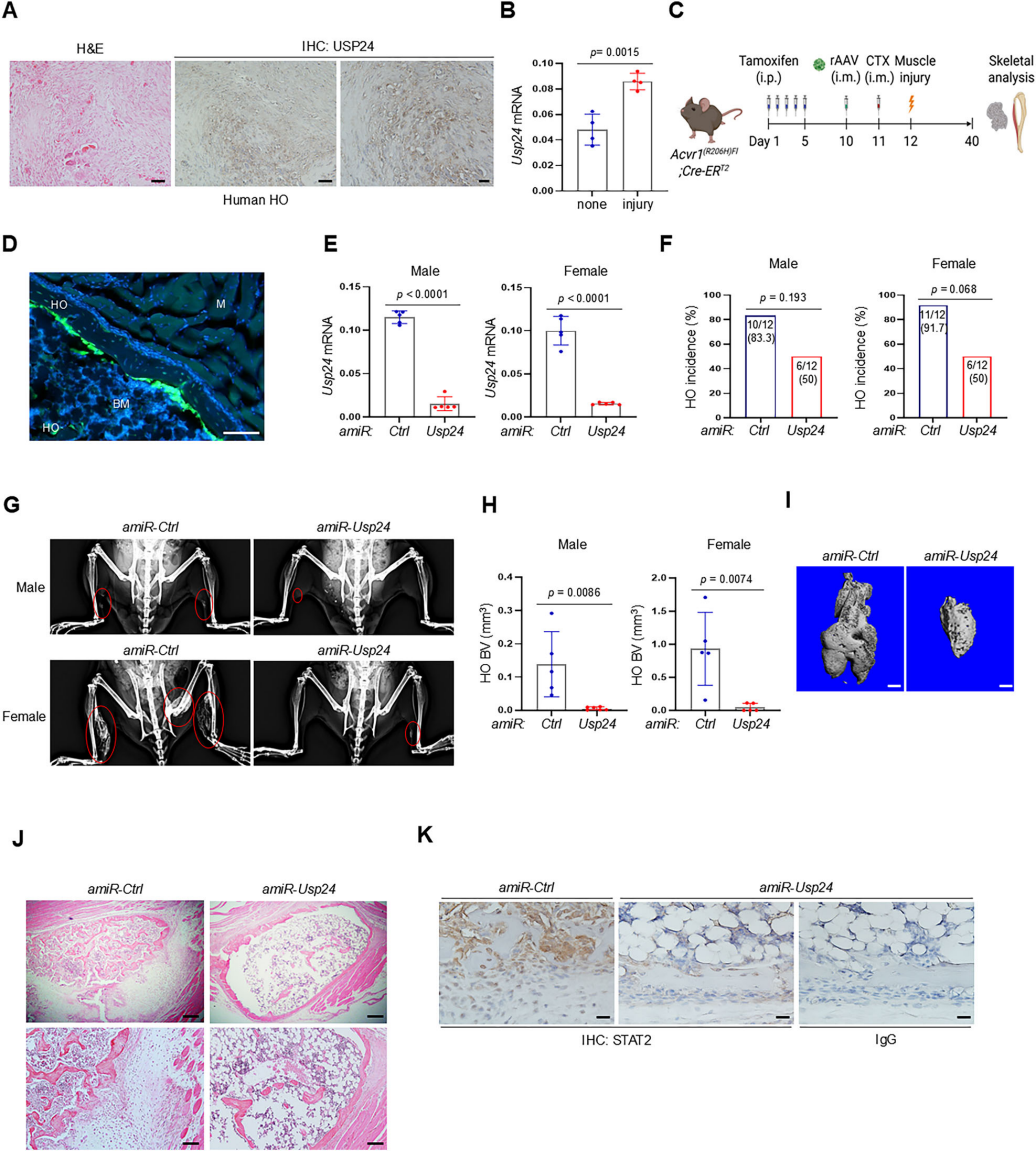
USP24 induces heterotopic ossification in vivo FOP mouse model. **A** H&E-stained sections (left) and IHC (middle and right) for USP24 in human tissue with heterotopic ossification (HO). Scale bar, 50 μm (left and middle) or 20 μm (right). **B** 8-week-old *Acvr1*^*(R206H)Fl*^*; Cre-ER*^*T2*^ mice were treated with intraperitoneal (i.p.) injection of tamoxifen for 5 days, and 3 days later, the pinch injury/cardiotoxin (CTX) injection was introduced to the gastrocnemius muscle. 24 h after the injury, the tibial muscle was dissected, and *Usp24* mRNA levels were assessed by qRT-PCR (*n* = 4). **C** Schematic diagram showing experimental strategy and plan. **D** 8-week-old *Acvr1*^*(R206H)Fl*^*;Cre-ER*^*T2*^ mice were treated with i.p. injection of tamoxifen for 5 days and 5 days later, 5 × 10^12^ vg/kg of rAAV9.egfp was injected into the gastrocnemius muscle (i.m.), followed by CTX injection and pinch injury. 28 days later, EGFP expression was assessed by fluorescence microscopy. Scale bar, 100 μm. HO, heterotopic ossification; BM, bone marrow; M, muscle. **E** 5 × 10^12^ vg/kg of rAAV9.egfp carrying *amiR-Ctrl* or *amiR-Usp24* was injected into 8-week-old *Acvr1*^*(R206H)Fl*^*;Cre-ER*^*T2*^ mice as described in Fig. 4C, and *Usp24* mRNA levels were assessed by qRT-PCR (*n* = 6 for male and *n* = 5 for female). **F** Incidence (%) of HO-positive mice. The number of HO-positive mice is shown as a proportion of the total mice in each group (*n* = 12 per group). In males, the incidence was 10/12 (83.3%) for the *amiR-Ctrl-* and 6/12 (50.0%) for the *amiR-Usp24*-treated group (*p* = 0.193). In females, 11/12 (91.7%) for the *amiR-Ctrl-* and 6/12 (50.0%) for the *amiR*-*Usp24*-treated group (*p* = 0.068). HO was detected by radiography and quantified by micro-CT. Representative radiographic images (**G**), quantified HO bone volume (BV, **H**, *n* = 5), and 3D reconstruction images (**I**) were shown. Scale bar, 1 mm (**I**). HO was assessed by H&E staining (**J**) and IHC for STAT2 (**K**) in rAAV-treated femurs. Scale bar, 250 μm (J, upper), 100 μm (J, lower), or 20 μm (**K**). IgG was used as a negative control. Data are representative of three (**A, D, G, I–K**). A two-tailed unpaired Student’s *t* test for comparing two groups (**B, E, H**; error bars, SD of biological replicates). For Fig. 4F, statistical significance was determined by Fisher’s exact test.

Inflammation and aberrant bone formation are key components of heterotopic ossification (HO), a form of pathologic bone formation. Our results indicate that USP24 is essential for physiological bone formation (Fig. 2), and previous studies have shown that USP24 can positively regulate inflammation through nuclear factor kappa-light-chain-enhancer of activated B cells (NF-κB) signaling [24]. Based on these observations, we hypothesized that USP24 mediates HO pathogenesis. To test this, we first examined its expression in human and mouse HO tissues. Immunohistochemistry revealed the localization of USP24 on the surface of heterotopic bone in human HO samples (Fig. 4A). Similarly, *Usp24* mRNA levels were markedly upregulated at injury sites in a mouse model of acquired HO (Fig. 4B). Therefore, we employed a mouse model of fibrodysplasia ossificans progressiva (FOP), a genetic HO. FOP patients have gain-of-function mutations in the bone morphogenetic protein (BMP) type I receptor (Activin A Receptor Type 1, ACVR1), and approximately 97% of FOP patients harbor a recurrent *ACVR1*^*R206H*^ mutation [25, 26]. FOP mice were generated by crossing the mice carrying a conditional knock-in allele of human *ACVR1*^*R206H*^ mutation (*ACVR1*^*Fl(R206H)*^) with *Cre-ER*^*T2*^ mice [27, 28]. Following 5 days of tamoxifen treatment, HO was induced by muscle injury using cardiotoxin (CTX) injection and pinch injury. rAAV vectors encoding either *amiR-Ctrl* or *amiR-Usp24* were delivered to the injured muscle. HO formation was assessed 28 days post-injury (Fig. 4C). EGFP expression confirmed rAAV-mediated gene delivery to HO lesions (Fig. 4D), and robust *Usp24* silencing was validated by qRT-PCR (Fig. 4E).

USP24 deficiency dramatically reduced both the incidence and severity of HO in male and female mice (Fig. 4F, G). Micro-CT analysis showed that *amiR-Usp24* treatment suppressed HO bone volume (BV) in the injured muscle (Fig. 4H, I). Histological analysis of hematoxylin and eosin (H&E)-stained sections revealed reduced endochondral ossification in *amiR-Usp24*-treated mice, whereas control-treated mice developed mature heterotopic bone composed of chondrocytes, bone marrow compartments, and bone scaffolds (Fig. 4J). Furthermore, IHC for STAT2 showed significantly decreased expression in HO lesions of *amiR-Usp24*-treated mice (Fig. 4K), implicating the possibility that the USP24-STAT2 signaling axis is critical for HO pathogenesis in FOP mice.

**Fig. 5:**
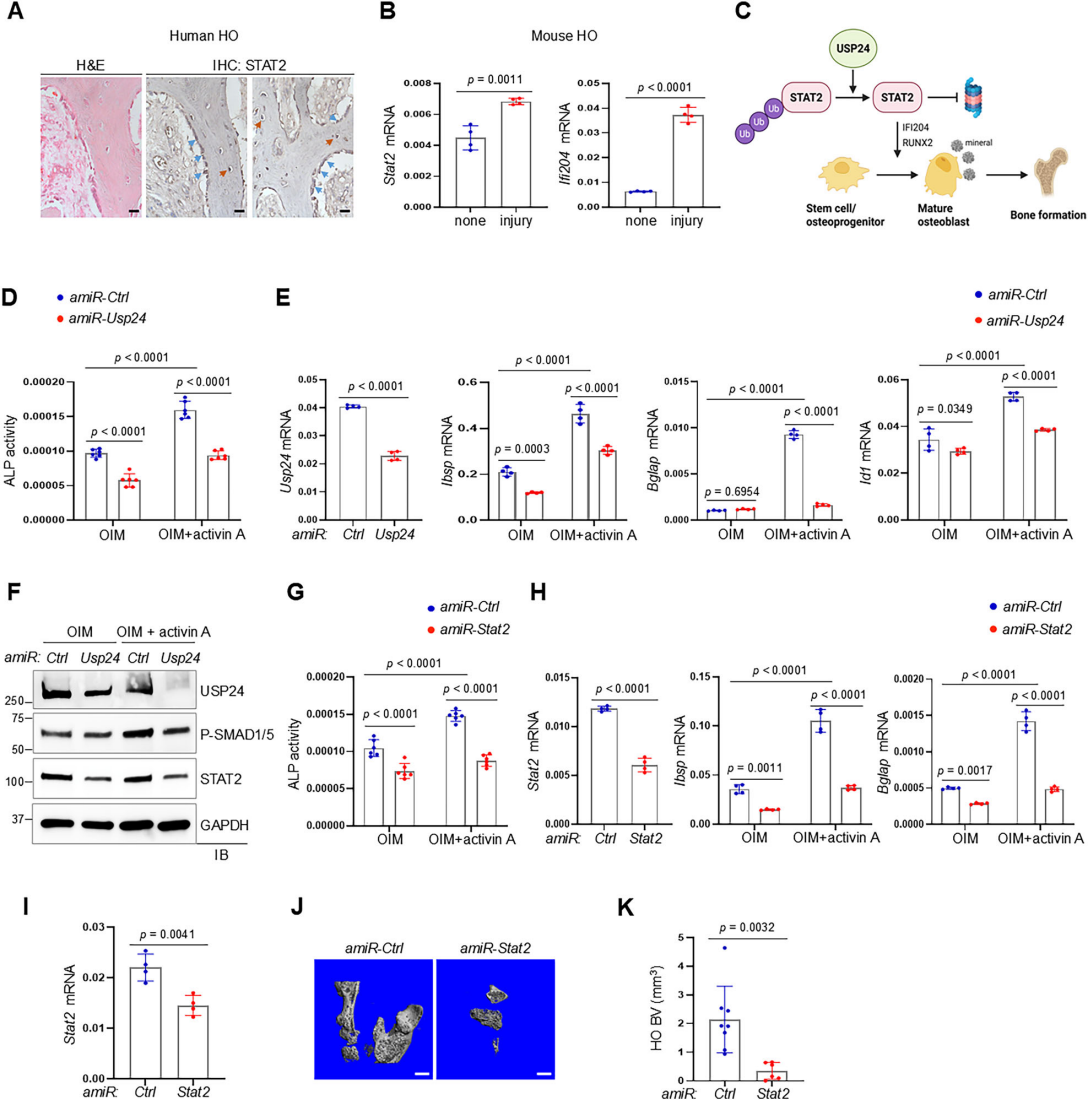
A USP24/STAT2 signal axis is required for HO formation. **A** H&E-staining (left) and IHC for STAT2 (right) in human HO tissue. Blue arrows indicate bone-lining cells; Orange arrows indicate bone-embedded osteocytes. Scale bar, 20 μm. **B** 8-week-old *Acvr1*^*(R206H)Fl*^*; Cre-ER*^*T2*^ mice were treated with i.p. injection of tamoxifen for 5 days, and 5 days later, the pinch injury and CTX injection were introduced to the gastrocnemius muscle. 24 h after injury, the tibial muscle was dissected, and *Stat2* and *Ifi204* mRNA levels were assessed by qRT-PCR (*n* = 4). **C** Schematic diagram illustrating the mechanisms by which USP24 promotes osteoblast differentiation through STAT2 stabilization. *Prrx1*^*+*^ osteogenic progenitors isolated from 4-week-old *Prrx1-Cre* (*Acvr1*^*WT*^) or *Acvr1*^*(R206H)Fl*^*;Prrx1-Cre* femurs, transduced with 5 × 10^10^ GCs of rAAV9.egfp carrying *amiR-Ctrl* or *amiR-Usp24*, and cultured in osteogenic induction medium (OIM) with or without activin A (50 ng/ml). ALP activity (**D**, *n* = 6) and mRNA levels of osteoblast genes (**E**, *n* = 4) were examined at day 6. Protein levels of USP24, phospho-SMAD1/5, and STAT2 were determined by immunoblotting at day 1 of osteogenic differentiation (**F**). *Prrx1*^*+*^ osteogenic progenitors isolated from 4-week-old *Prrx1-Cre* (*Acvr1*^*WT*^) or *Acvr1*^*(R206H)Fl*^*;Prrx1-Cre* femurs were transduced with 5 × 10^10^ GCs of rAAV9.egfp carrying *amiR-Ctrl* or *amiR-Stat2* and cultured in OIM with or without activin A (50 ng/ml). ALP activity (**G**, *n* = 6) and mRNA levels of osteoblast genes (**H**, *n* = 4) were examined at day 6. **I** 5 × 10^12^ vg/kg of rAAV9.egfp carrying *amiR-Ctrl* or *amiR-Stat2* was injected into 8-week-old *Acvr1*^*(R206H)Fl*^*;Cre-ER*^*T2*^ mice as described in Fig. 4C, and *Stat2* mRNA levels were assessed by qRT-PCR (*n* = 4). HO formation was assessed by micro-CT. 3D reconstruction images (**J**) and quantified HO bone volume (BV, **K** *n* = 8 for *amiR-Ctrl* and *n* = 6 for *amiR-Stat2*) were shown. Scale bar, 1 mm (**J**). Data are representative of three (**A, F, J**). A two-tailed unpaired Student’s *t* test for comparing two groups (**B, D, E, G–I, K**; error bars, SD of biological replicates).

To investigate the role of STAT2 in HO formation, we performed IHC for STAT2 in human HO tissues, demonstrating that STAT2 was highly expressed in bone-lining osteoblasts as well as osteocytes within heterotopic bone matrix (Fig. 5A). Consistently, mRNA levels of *Stat2* and IFN-activated gene 204 (*Ifi204*) were upregulated at injury sites in a mouse model of acquired HO (Fig. 5B). Previous studies have shown that STAT2 cooperates with other STAT proteins or IRF9 to induce transcription of *IFI204* (also known as p204) [22, 23], which functions as a coactivator of RUNX2 and positively regulates osteoblast differentiation²⁵. Knockdown of *Stat2* reduced *Ifi204* expression in osteoblasts (Supplementary Fig. S7A) and concomitantly decreased RUNX2 transcriptional activity (Supplementary Fig. S7B), suggesting that STAT2 may promote osteoblast differentiation and bone formation through IFI204-mediated RUNX2 regulation (Fig. 5C).

To determine the molecular mechanism by which STAT2 regulates RUNX2 in osteoblasts, we performed co-immunoprecipitation analysis in osteoblasts, demonstrating no detectable interaction between STAT2 and RUNX2 (Supplementary Fig. S7C). Next, we examined the possibility that STAT2 directly regulates RUNX2 transcription by binding to the *RUNX2* promoter. Using publicly available chromatin immunoprecipitation sequencing (ChIP-seq) datasets, we identified two putative STAT2 binding sites located in the distal promoter, at approximately –134,706 bp (GSE97661) and -99,454 bp (GSE128111) relative to the RUNX2 transcription start site (TSS). In contrast, STAT1 and STAT3 bind within the proximal promoter regions of *RUNX2*, with STAT occupying from -791 bp to -185 bp (GSE100566) and STAT3 spanning -888 bp to -269 bp across multiple datasets (GSE85579, GSE152203, GSE85579, GSE79707, GSE152203, GSE85579, and GSE115597). These findings suggest that STAT2 may modulate RUNX2 transcription, potentially as part of a heteromeric transcriptional complex with other STAT family members (STAT1, STAT3), and/or the co-activator IFI204. Notably, *IFI204* transcription can be regulated by STAT2 together with other STAT family members and IRF9.

In FOP patients, activin A binds to ACVR1, activates BMP signaling and induces HO formation [29]. To determine whether the USP24-STAT2 signaling axis regulates activin A-induced BMP signaling in osteoblasts that mediates HO pathogenesis, primary BMSCs from FOP mice were transduced with rAAV vectors encoding either *amiR-Ctrl* or *amiR-Usp24* and cultured under osteogenic conditions with or without activin A. As expected, activin A markedly increased ALP activity, expression of osteoblast markers (*Ibsp* and *Bglap*), and BMP target gene (*Id1*), all of which were attenuated by *Usp24* knockdown (Fig. 5D, E). USP24 deficiency also reduced phosphorylation of SMAD1/5, key BMP downstream effectors, as well as STAT2 protein levels (Fig. 5F). Similarly, *Stat2* knockdown decreased ALP activity and osteoblast gene expression under activin A-induced osteogenic conditions (Fig. 5G, H). These results highlight the critical role of the USP24-STAT2 signaling axis in Activin A-induced BMP signaling and osteogenic differentiation of FOP BMSCs. In addition to its role in Activin A signaling and osteogenic differentiation, STAT2 may also contribute to HO formation by modulating chemokine and IFN signaling pathways, which mediate injury-induced immune responses and the subsequent recruitment of inflammatory cells [30, 31]. Specifically, C-X-C motif chemokine 10 (CXCL10) [32], IFN regulatory factor 7 (IRF7)^33^, and IFN-stimulated gene 15 (ISG15)^34^ have been implicated in this process during HO development. Given that knockdown of *Stat2* in FOP osteoblasts reduced the expression of these inflammatory mediators (Supplementary Fig. S8), STAT2-mediated regulation of ISGs may contribute to the initiation of HO pathogenesis in FOP. To evaluate the therapeutic potential of *Stat2* silencing in vivo, rAAV vectors encoding *amiR-Ctrl* or *amiR-Stat2* were delivered to FOP mice as described in Fig. 4C. Robust silencing of *Stat2* in HO lesions was confirmed (Fig. 5I). Consistent with USP24 deficiency, rAAV-mediated silencing of *Stat2* significantly suppressed HO formation in FOP mice, as assessed by micro-CT analysis (Fig. 5J, K). These results indicate that the USP24-STAT2 axis mediates chemokine/IFN and Activin A signaling pathways, which are critical for trauma-induced HO in FOP mice.

## Discussion

Our data demonstrates that USP24 promotes bone formation by stabilizing STAT2 under both physiologic and pathologic conditions. Loss of USP24 impairs osteoblast differentiation and bone formation, whereas USP24 deficiency in osteoclasts has no effect on their differentiation. Mechanistically, USP24 stabilizes STAT2 by preventing ubiquitin-dependent proteasomal degradation (Fig. 5C) USP24 interacts with STAT2 in osteoblasts, and USP24 deficiency reduces STAT2 protein levels without affecting *Stat2* mRNA expression, STAT2 phosphorylation, and its transcription activity. Consistently, rAAV-mediated silencing of *Usp24* using artificial miRNA lowered STAT2 protein levels in osteoblasts during skeletal development and homeostasis. This reduction was particularly pronounced during FOP progression, highlighting the USP24–STAT2 signaling axis as a bone anabolic pathway in both normal and pathological bone formation.

HO, particularly in the context of FOP, represents a devastating form of pathological bone formation. Current therapeutic approaches—such as bisphosphonates, external beam radiation, and non-steroidal anti-inflammatory drugs (NSAIDs)—are limited by suboptimal efficacy, potential toxicities, and their tendency to impair the healing of concurrent skeletal injuries [33, 34]. In FOP, several agents are under clinical evaluation, including a retinoic acid receptor γ agonist (palovarotene), an anti-activin A antibody (REGN 2477), an immunosuppressant (rapamycin), and ACVR1 kinase inhibitors (IPN60130); however, their clinical use remains restricted due to potential adverse effects [35, 36]. Thus, there is an urgent need for safer and more effective therapies. Here, we identify the USP24-STAT2 signaling axis as a druggable target for HO in FOP. rAAV-mediated silencing of *Usp24* or *Stat2* effectively suppressed trauma-induced HO in FOP mice, providing proof-of-principle that rAAV-mediated inhibition of the USP24-STAT2 axis has therapeutic potential for suppressing HO in FOP.

The rAAV vectors have been extensively used in gene therapy due to their high transduction efficiency, long-term expression, relatively low immunogenicity, and favorable safety profile [37, 38]. However, high-dose administration of rAAV-delivered shRNAs causes cytotoxicity by disrupting the endogenous RNA interference (RNAi) machinery [39, 40]. Therefore, we employed an artificial microRNA (amiRNA) strategy by embedding the guide strand targeting *Usp24* or *Stat2* into a mouse miR-33-derived scaffold [41, 42]. Previously, we demonstrated that a single systemic dose of rAAV9 vector at postnatal day 0 (10^11^ genome copies) or at the age of 3-month-old (5 × 10^13^ vg/kg) effectively transduces the liver, heart, muscle, lung, kidney, and bone, but not the brain and spleen, following intravenous injection [28, 43]. However, rAAV9 has been reported to be inefficient at transducing immune cells [44]. In this context, despite the multifaceted roles of USP24 and STAT2 in systemic immunity and metabolism, histopathological analysis revealed little to no abnormalities in the heart, liver, lung, kidney, and spleen of rAAV-treated mice (Supplementary Figs. S2 and S3), indicating that AAV-mediated silencing of *Usp24* or *Stat2* does not induce any toxicity in non-bone tissues. Future vector improvements to selectively target osteoblast lineage cells, such as using osteoblast-specific promoters or engineered AAV capsids, will enable more precise bone-targeting rAAV vectors capable of delivering therapeutic genes without off-target effects in non-skeletal tissues. Nonetheless, the long-term therapeutic outcomes and safety evaluation also require further investigation, given the broad roles of USP24 and STAT2 in cellular processes beyond osteogenesis.

USP24 has been implicated in diverse cellular functions, particularly in cell survival and apoptosis. Given its overexpression in many cancer cells, USP24 acts as a molecular rheostat that can either promote survival or trigger death depending on the cellular context and its specific substrates. For example, USP24 exerts anti-apoptotic effects by deubiquitinating and stabilizing myeloid cell leukemia-1 (MCL-1), an anti-apoptotic member of the B-cell lymphoma 2 (BCL-2) family, protecting cancer cells from apoptosis and contributing to chemotherapy resistance [45]. Additionally, USP24 stabilizes programmed cell death protein 1 (PD-1), helping cancer cells evade immune-mediated apoptosis by T cells [46]. On the other hand, USP24 functions as a pro-apoptotic regulator in response to DNA damage or oxidative stress. By stabilizing p53, USP24 triggers the transcription of pro-apoptotic genes, such as BCL2-associated X (*BAX*), ensuring that damaged cells undergo apoptosis rather than continue to divide [17]. Additionally, USP24 indirectly modulates cell fate by stabilizing unc-51-like autophagy activating kinase 1 (ULK1), a master regulator of autophagy and mitophagy [47]. By maintaining mitochondrial integrity via mitophagy, USP24 prevents the inadvertent release of pro-apoptotic factors into the cytoplasm. Consequently, small-molecule inhibitors targeting USP24 are being explored as a strategy to overcome therapy-induced drug resistance [48]. Given this context-dependent regulatory capacity, it is plausible that USP24 similarly functions as a molecular rheostat in the skeletal system, determining osteoblast fate by modulating the balance between survival and apoptosis. Although we employed rAAV-mediated silencing of *Usp24* in the skeleton, this approach may also hold promise in oncology, offering enhanced precision, cell-type specificity, and durable efficacy.

Here, we identify USP24-mediated stabilization of STAT2 as a novel therapeutic target for pathologic bone formation. The mechanisms regulating STAT2 protein stability remain poorly understood. To date, the only known factor promoting STAT2 degradation is the E3 ubiquitin ligase F-box and WD repeat domain-containing-7 (FBXW7), which ubiquitinates STAT2 and drives its proteasomal degradation in melanoma cells [49]. Notably, FBXW7 has also been reported to act as a negative regulator of osteoblast formation [50, 51]. However, a DUB for STAT2 has not previously been defined. Our findings establish USP24 as the DUB that stabilizes STAT2 in osteoblast-lineage cells to promote bone formation.

Although *Stat2*-deficient mice were previously reported to develop without overt skeletal abnormalities [52], our data uncover a previously unrecognized anabolic role for STAT2 in bone formation, particularly under pathologic conditions. RUNX2, a master transcription factor essential for osteoblast lineage commitment and differentiation, is required for both physiologic and pathologic bone formation [14, 53–55]. We hypothesize that STAT2 modulates RUNX2 transcription, potentially as part of a heteromeric transcriptional complex with other STAT family members (STAT1, STAT3), and/or the co-activator IFI204, thereby promoting osteoblast development and bone formation (Fig. 5C). During HO pathogenesis in FOP, STAT2 not only mediates Activin A signaling and osteogenic differentiation of FOP osteoblasts, but also induces injury-induced immune responses and the subsequent recruitment of inflammatory cells by modulating chemokine and IFN signaling pathways. In addition, STAT2 may contribute to senescence-associated programs, further linking inflammatory signaling to osteogenic remodeling during HO. In chronic inflammatory environments, where sustained IFN signaling is intrinsically linked to senescence-like phenotypes, the USP24-STAT2 axis may orchestrate a pro-osteogenic microenvironment by modulating the balance between cell survival, senescence, and differentiation. Consequently, elucidating how STAT2, in coordination with its binding partners, orchestrates osteogenic programming remains a critical avenue for understanding physiologic and pathologic bone formation.

## Materials and methods

### Cell culture, plasmids, recombinant proteins, and antibodies

HEK293T cells and C3H10T1/2 cells were purchased from ATCC and grown in Dulbecco’s modified Eagle’s medium (DMEM) (Corning, 10-013-CV) supplemented with 10% fetal bovine serum (FBS, Corning, 35-015-CV), 2 mM L-glutamine (Corning, 25030081), 1% nonessential amino acids (Corning, 25-025-CI), and 1% penicillin/streptomycin (Corning, 30-002-CI). All cell lines were regularly tested for mycoplasma contamination. Human bone marrow-derived mesenchymal stem cells (BMSCs) were purchased from ScienCell and were maintained in the growth medium (7501) and cultured in osteogenic medium (7531) according to the manufacturer’s instructions. The construct for Flag-*USP24* was generated as previously described [17]. The constructs for STAT1 and STAT3 were deposited to Addgene by Steven Johnson (#11987) and Geert van den Bogaart (#111934), respectively. Myc-tagged *STAT2* construct (HG10547-NM) was obtained from Sino Biological Inc. The construct for HA-ubiquitin was deposited to Addgene by Edward Yeh (#18712), and the plasmid for His-ubiquitin was generated by subcloning. The construct for interferon-stimulated response elements (ISRE)-luc was deposited to Addgene by a gift from Promega Corporation (#236834). Recombinant activin A (338-AC) and interferon alpha-2 (93613) were purchased from R&D Systems and Abclonal, respectively. Antibodies specific to STAT1 (Abclonal, A19563), STAT2 (Cell signaling, 4594), P-STAT2 (Abclonal, AP0284), STAT3 (Cell signaling, 9139), USP24 (Proteintech, 13126-1-AP), P-SMAD1/5 (Cell signaling, 9516), and GAPDH (EMD Millipore, CB1001) were used according to manufacturers’ instructions. Horseradish peroxidase (HRP)-conjugated anti-rabbit (sc-2357) and anti-mouse (10735086001) secondary antibodies were purchased from Santa Cruz Biotechnology.

### rAAV vector design and production

Bone-targeting rAAV9 vectors were generated as described in previous studies [42, 56]. DNA sequences for *amiR-33-Ctrl*, *amiR-33-Usp24*, and *amiR-33-Stat2* were synthesized as gBlocks and cloned into the intronic region of the pAAVsc-*CB6-*enhanced green fluorescent protein (*Egfp*) plasmid at the restriction enzyme sites (PstI and BglII) [57]. The pAAV-*amiR-Ctrl*, pAAV-*amiR-Usp24*, and pAAV*-amiR-Stat2* constructs were packaged into the rAAV9 capsid. rAAV production was performed by transient transfection of HEK293 cells, purified by CsCl sedimentation, and titrated by droplet digital PCR (ddPCR) on a QX200 ddPCR system (Bio-Rad). The sequences of gBlocks and oligonucleotides for ddPCR are listed in Supplementary Table S1.

### Mice

C57BL/6 mice were purchased from the Jackson Laboratory and used to test the *Usp24* silencing effect on skeletal development and homeostasis. *amiR-Ctrl* or *amiR-Usp24*-expressing rAAV vectors were injected into postnatal day 0 (neonate) pups via the facial vein or 3-month-old mice intravenously (i.v.). To test *Usp24* or *Stat2* silencing effects on HO formation in FOP, mice harboring a conditional *Acvr1*^*R206H*^ knock-in allele (*Acvr1*^*(R206H)Fl*^) [27] were kindly gifted from the International FOP Association via Dr Daniel Perrien (Emory University) and maintained on a C57BL/6 J background. The target construct described in the previous study [28] was inserted into the locus of mouse *Acvr1*. Wildtype mouse exons 5-10, neomycin-resistant gene (neo cassette), one F3 site, and one loxP site are deleted by *Cre* recombinase, resulting in expression of human cDNA exons 6-11 harboring the R206H mutation and EGFP. *Acvr1*^*(R206H)Fl*^ mice were crossed with *Cre-ER*^*T2*^ mice (*Acvr1*^*(R206H)Fl*^*; Cre-ER*^*T2*^) where tamoxifen-induced expression of *Cre* recombinase mediates *Acvr1*^*R206H*^-driven HO; *Prrx1-Cre* mice (*Acvr1*^*(R206H)Fl*^; *Prrx1-Cre*), where expression of *Cre* recombinase in *Prrx1*^*+*^ skeletal progenitors in the limb mesenchyme mediates *Acvr1*^*R206H*^-driven HO. *Cre-ER*^*T2*^ and *Prrx1-Cre* mice were purchased from Jackson Laboratory and maintained on C57BL/6 J background. Mouse genotypes were determined by PCR using tail genomic DNA. Primer sequences are available upon request. Control littermates were used and analyzed in all experiments. All animals were used in accordance with the NIH Guide for the Care and Use of Laboratory Animals and were handled according to protocols approved by the University of Massachusetts Chan Medical School on Animal Care (IACUC, Approval #202200036).

### Trauma-induced heterotopic ossification (HO) mouse model

6-week-old *Acvr1*^*(R206H)Fl*^*; Cre-ER*^*T2*^ mice were daily treated with intraperitoneal (i.p.) injection of tamoxifen (10 mg/kg) for five days, and three days later, 5 × 10^12^ vg/kg of rAAV9 vectors carrying *amiR-ctrl* (scrambled control amiR), *amiR-Usp24* or *amiR-Stat2* were injected into the muscle of hindlimbs. 1 μM cardiotoxin (CTX)/pinch injury was employed in the gastrocnemius muscle three days after rAAV injection.

### Micro-CT and radiography

For skeletal analysis, sex-matched and control littermates were used and randomly assigned in all experiments. Micro-CT was used for the qualitative and quantitative assessment of trabecular and cortical bone microarchitecture, performed by an investigator blinded to the groups of animals under analysis. Femurs excised from the indicated mice were scanned using a micro-CT 35 (Scanco Medical) with a spatial resolution of 7 μm (Scanning parameters: 55 kVp and 114 mA) according to guidelines [58]. For trabecular bone analysis of the distal femur, an upper 2.1 mm region beginning 280 μm proximal to the growth plate was contoured (Segmentation: sigma 0.8, support 1, threshold 280). For cortical bone analysis of the femur, a midshaft region of 0.6 mm in length was used (Segmentation: sigma 0.8, support 1, threshold 350). Micro-CT scans of heterotopic ossification in muscle were performed using isotropic voxel sizes of 12 μm. 3D reconstruction images were obtained from contoured 2D images by methods based on distance transformation of the binarized images. All images presented are representative of the respective groups (*n* > 5).

Radiographic images of hindlimbs were taken by the Trident Specimen Radiography System (Hologic, Inc.). The X-ray beam intensity was set to 1 mA 28-30 kV with automatic exposure control (AEC) for consistent image acquisition (*n* > 5).

### Histology, histomorphometry, immunohistochemistry, and immunofluorescence

All experiments for histology, histomorphometry, immunohistochemistry, and immunofluorescence were performed based on previous publications with modifications [14, 59]. Briefly, for histological analysis, femurs were dissected from the mice, fixed in 10% neutral buffered formalin for 2 days, and decalcified by every 2-day changes of 0.5 M tetrasodium EDTA for 3 to 4 weeks. Tissues were dehydrated by passage through an ethanol series, cleared twice in xylene, embedded in paraffin, and sectioned at 7 μm thickness along the coronal plate from anterior to posterior. Decalcified femoral sections were stained with hematoxylin and eosin (H&E) or tartrate-resistant acid phosphatase (TRAP).

For histomorphometric analysis, 25 mg/kg calcein (Sigma, C0875) and 50 mg/kg alizarin-3-methyliminodiacetic acid (Sigma, A3882) dissolved in 2% NaHCO₃ solution were intraperitoneally injected into 4-week-old male mice at 3-day intervals. After 2 days of fixation in 10% neutral buffered formalin, undecalcified femur samples were embedded in methylmethacrylate. A region of interest was defined in the trabecular bone in the metaphysis, and bone formation rate/bone surface (BFR/BS), mineral apposition rate (MAR), osteoblast surface/bone surface (Ob.S/BS) were measured using a semiautomatic analysis system (OsteoMetrics, Atlanta, GA, USA). Measurements were taken on two sections/sample (separated by ~25 μm) and summed up prior to normalization to obtain a single measure/sample in accordance with ASBMR standards [60, 61]. This methodology has undergone extensive quality control and validation, and the results were assessed by a research specialist in a blinded fashion.

For immunohistochemistry (IHC), paraffin sections were deparaffinized and hydrated. Citrate-based solutions (VECTOR, H3300) and 3% H_2_O_2_/MeOH were used for antigen retrieval and quenching, respectively. After sections were incubated with antibodies specific to USP24 (1:100, Novus Biologicals, NBP1-82942) or STAT2 (1:100, Abclonal, A14995) for 40 min at 37 °C, and secondary antibodies for 20 min at 37 °C. Subsequently, they were incubated in streptavidin-horseradish peroxidase (SA-HRP) for 16 min at 37 °C and then in 3,3′-Diaminobenzidine (DAB) + H_2_O_2_ substrate for 8 min, followed by hematoxylin and bluing reagent counterstain at 37 °C.

For immunofluorescence, femoral bone was fixed with 4% paraformaldehyde (PFA) for 2 days and decalcified in 0.5 M tetrasodium EDTA solution for 10 days. Semi-decalcified samples were infiltrated with 25% sucrose phosphate for 4 days. All samples were embedded in a 50/50 mixture of 25% sucrose solution and optimal cutting temperature (OCT, a water-soluble blend of glycols and resins) compound (Sakura) and cut into 12-μm-thick sagittal sections using a cryostat (Leica).

### Osteoblast differentiation

Primary calvarial osteoblasts (COBs) were isolated from the calvarium of 5-day-old C57BL/6 neonates using collagenase type II (50 mg/ml, Worthington, LS004176)/dispase II (100 mg/ml, Roche, 10165859001). COBs were maintained in α-MEM medium (Corning, 10-022-CV) containing 10% FBS (Corning), 2 mM L-glutamine (Corning), 1% penicillin/streptomycin (Corning), and 1% nonessential amino acids (Corning). Mouse BMSCs were isolated from crushed long bones of 8-week-old mice and cultured under non-osteogenic medium (α-MEM medium, 10% FBS, 2 mM L-glutamine, 1% nonessential amino acids, and 1% penicillin/ streptomycin). For osteogenic differentiation, ascorbic acid (200 μM, Sigma, A8960) and β-glycerophosphate (10 mM, Sigma, G9422) were added to non-osteogenic medium. For alkaline phosphatase (ALP) staining, osteoblasts were fixed with 10% neutral formalin buffer and stained with the solution containing Fast Blue (Sigma, FBS25) and Naphthol AS-MX (Sigma, 855). Alternatively, for the ALP activity assay, osteoblasts were incubated with Alamar Blue solution (Invitrogen, DAL1100) to check cell viability. Subsequently, cells were washed with phosphate-buffered saline (PBS) and incubated in a solution containing 6.5 mM Na_2_CO_3_, 18.5 mM NaHCO_3_, 2 mM MgCl_2_, and phosphatase substrate (Sigma, S0942), and ALP activity was measured using a spectrometer. To assess extracellular matrix mineralization in mature osteoblasts, cells were washed twice with PBS and fixed in 70% EtOH for 15 min at room temperature. Fixed cells were washed twice with distilled water and then stained with a 2% alizarin red solution (Electron Microscopy Sciences, 26206-01) for 5 min. Cells were then washed three times with distilled water and examined for the presence of calcium deposits. Mineralization was quantified using the acetic acid extraction method [62].

### Osteoclast differentiation

For osteoclast culture, bone marrow-derived monocytes (BMMs) were collected by flushing bone marrow cells from the femur and tibia of 8-week-old C57BL/6 mice. Cells were plated, and the next day, non-adherent cells were transferred to a new plate and cultured in the presence of M-CSF (30 ng/ml, R&D Systems, 416-ML). When cells reached 80% confluency, they were treated with M-CSF (30 ng/ml) and RANKL (10 ng/ml, R&D Systems, 462-TEC) to induce osteoclast differentiation. TRAP staining was performed according to the manufacturer’s protocol (Sigma, 387 A). For the TRAP activity assay, 50 μl of culture supernatants collected from the BMM culture were incubated with a mixture of 0.1 M acetate solution (Sigma, 3863), 90 mM C_4_H_4_Na_2_O_6_, and 7.6 mM p-nitrophenyl phosphate (p-NPP) at 37 °C for 1 h. After that, 3 N NaOH was added, and the solution was measured at 405 nm using a spectrometer.

### Human subject

De-identified heterotopic bone samples were obtained from human patients in Yonsei University Severance Hospital, Korea, under institutional review board approval (IRB No.4-2017-1223). Informed consent was obtained from all subjects. The individuals included three patients (one male and two females, all previously healthy, nonsmoking individuals; ages ranged from 29 to 67 years) with pain or decreased hip movement. The specimens that showed spontaneous HO radiographically and pathologically were used for histology and IHC.

### Quantitative RT-PCR, immunoblotting, and pull-down assay

To prepare bone RNA samples from mouse limbs, the hindlimbs were dissected, and skin/muscle tissues were removed. The remaining tibias were chopped and homogenized. Total RNA from cells or tissues was extracted using QIAzol (Qiagen, 79306), and cDNA was synthesized using the High-Capacity cDNA Reverse Transcription Kit from Applied Biosystems. Quantitative RT-PCR (qRT-PCR) was performed using SYBR® Green PCR Master Mix (Bio-Rad, 1725121) with the Bio-Rad CFX Connect Real-Time PCR detection system. mRNA levels were normalized to the housekeeping gene Ribosomal protein, large, P0 (*Rplp0*). The primers used for PCR are described in Supplementary Table S2.

To examine the interaction between USP24 and STAT2, C3H10T1/2 cells were transfected with 1 μg of control or Flag-*USP24* with HA-ubiquitin DNA constructs using the Effectene transfection reagent (Qiagen). After 48 h, cells were treated with 10 μM MG132 for 6 h, and cell lysates were prepared in lysis buffer (50 mM Tris-HCl [pH 7.8], 150 mM NaCl, 1% Triton X-100, 1 mM dithiothreitol [DTT], 0.2% Sarkosyl acid, and protease inhibitor cocktail [Sigma, P8340]). Cell lysates were incubated with 40 μl of Flag-conjugated agarose (Sigma, A2220) at 4 °C overnight (12-16 h) and subjected to SDS-PAGE. Protein samples transferred to Immobilon-P membranes (Millipore) were immunoblotted with the indicated antibodies, then developed using ECL (Thermoscientific). To examine endogenous interaction between RUNX2 and STAT2, cell lysates were prepared from mouse COBs using the lysis buffer described above. Immunoblotting with an antibody specific to GAPDH was used as a loading control. All primary antibodies used for immunoblotting were diluted (1:1000) in 1X Tris Buffered Saline with Tween 20 (TBST) solution (137 mM Sodium Chloride, 20 mM Tris, 0.1% Tween-20, pH 7.4) that contained 5% bovine serum albumin (BSA, Roche, 10735086001). All secondary antibodies for immunoblotting were diluted (1:1000) in 1X TBST buffer that contained 5% skimmed milk. All uncropped, unmodified immunoblot images are included in the Supplementary material.

### Mass spectrometry

Plasmids expressing control (pcDNA3.1) or Flag-*USP24* were transfected into C3H10T1/2 cells using the Effectene transfection reagent. 48 h later, its binding proteins were co-immunoprecipitated with Flag-conjugated agarose, eluted with excessive amounts of Flag peptide (Sigma, F3290), and the eluates were subjected to mass spectrometry.

### Deubiquitination assay

To test the ability of USP24 to deubiquitinate ubiquitinated STAT proteins, a plasmid expressing *STAT1, STAT2*, or *STAT3* (1 μg) was transfected into HEK293T cells along with a His-ubiquitin-expressing plasmid (1 μg) in the absence or presence of a *USP24*-expressing plasmid (1 μg). 48 h later, cells were treated with 10 μM MG132 for 6 h, lysed and sonicated in denaturation buffer (8 M urea, 50 mM Tris pH 8.0, 1.0% Triton X-100, 10 mM imidazole, 10 mM β-mercaptoethanol), and pulled down with 40 μl of Ni-NTA beads (Qiagen, 30210) at room temperature overnight. The pull-down samples were subjected to SDS-PAGE and then immunoblotted with anti-STAT1, STAT2, or STAT3 antibodies.

### Luciferase reporter assay

A RUNX2-responsive reporter gene (OG2-luc) or an ISRE-luc construct, together with a *Renilla* luciferase control vector, was transfected into C3H10T1/2 cells using the Effectene transfection reagent (Qiagen). After 48 h, a dual luciferase assay was performed according to the manufacturer’s protocol (Promega, E2920), and firefly luciferase activity (OG2 or ISRE) was normalized to *Renilla* luciferase activity.

### Statistics and reproducibility

All experiments were performed at least twice or three times; for IHC, histological staining, and immunoblotting, representative images are shown. All data are shown as the mean ± Standard Deviation (SD). We first performed the Shapiro-Wilk normality test to check the normal distributions of the groups. If normality tests passed, a two-tailed, unpaired Student’s *t* test was used; if normality tests failed, a Mann-Whitney test was used for the comparisons between two groups. For the comparisons of three groups, we used one-way ANOVA if the normality tests passed, followed by Tukey’s multiple comparison test for all pairs of groups. The GraphPad PRISM software (ver.10.6.0, La Jolla, CA) was used for statistical analysis. *p* < 0.05 was considered statistically significant.

## Supplementary information

Supplementary information_clean

Uncropped immunoblot images

## Data Availability

Data supporting the findings of this manuscript are available from the corresponding author upon reasonable request.
