# Supplementary material for: USP24-dependent STAT2 stabilization mediates physiologic and pathologic bone formation
Source: Cell Death Dis. 2026 May 30;17(1):675. doi: 10.1038/s41419-026-08888-7 (PMC13433775; doi:10.1038/s41419-026-08888-7)
Supplement: Supplementary file 1 [file 41419_2026_8888_MOESM1_ESM.pdf]

Supplementary information

USP24-dependent STAT2 stabilization mediates physiologic and pathologic bone formation

Jung-Min Kim[1*], Yeon-Suk Yang[2,3], Jun Xie[2,4,5], Chan Mi Kang[1], Yeong-Chan Cho[1], Kwang Hwan Park[6], Feng Gong[7], Tadatoshi Sato[3,4,8], Beom-Jik Kim[1], Guangping Gao[2,4,5,8], and Jae-Hyuck Shim[2,3,4,8*]

[1]Department of Biology, College of Sciences, Kyung Hee University, Seoul, 02447, South Korea

[2]Department of Genetic and Cellular Medicine, [3]Department of Medicine, UMass Chan Medical School, Worcester, Massachusetts, USA

[4]Horae Gene Therapy Center, UMass Chan Medical School, Worcester, Massachusetts, USA

[5]Viral Vector Core, UMass Chan Medical School, Worcester, Massachusetts, USA

[6]Department of Orthopaedic Surgery, Yonsei University College of Medicine, Seoul, South Korea

[7]Department of Biochemistry and Molecular Biology, University of Miami Miller School of Medicine, Miami, FL 33136, USA

[8]Li Weibo Institute for Rare Diseases Research, UMass Chan Medical School, Worcester, MA 01605, USA

*To whom correspondence should be addressed.

Jung-Min Kim

Department of Biology, College of Sciences,

Kyung Hee University

E-mail: jungminkim@khu.ac.kr ; jmhere15@gmail.com

and

Jae-Hyuck Shim

Department of Genetic and Cellular Medicine,

UMass Chan Medical School

366 Plantation street, DiMare N9-1029, Worcester, MA 01605

E-mail: jaehyuck.shim@umassmed.edu

Running Title: USP24-STAT2 pathway in bone formation

Supplementary Table S1: rAAV vector sequences

**Construct**	**Sequence**
amiR-33-Ctrl (*amiR-Ctrl*)	tttgtcttttatttcaggtcccagatctagggctctgcgtttgctccaggtagtccgctgctcccttgggcctgggccc actgacagccctggtgcctctggccggctgcacacctcctggcgggcagctgtgtacaaactacttgagagcag gtgttctggcaatacctgcctgctctgtaatagtttgtacacggaggcctgccctgactgcccacggtgccgtggc caaagaggatctaagggcaccgctgagggcctacctaaccatcgtggggaataaggacagtgtcacccctgca ggggatccggtggtggtgcaaatca
EGFP-probe	6fam-cgcgatcacatggtcctgctgg-tamra
amiR-33-mUsp24 (*amiR-Usp24*)	gatctagggctctgcgtttgctccaggtagtccgctgctcccttgggcctgggcccactgacagccctggtgcctc tggccggctgcacacctcctggcgggcagctgtgtttctgcctaaagaatccagctgttctggcaatacctggctg gattgatcaggcagaaacacggaggcctgccctgactgcccacggtgccgtggccaaagaggatctaagggc accgctgagggcctacctaaccatcgtggggaataaggacagtgtcaccccccctgca
amiR-33-mStat2 (*amiR-Stat2*)	agggctctgcgtttgctccaggtagtccgctgctcccttgggcctgggcccactgacagccctggtgcctctggc cggctgcacacctcctggcgggcagctgtggttaatcggccaaccagtccttgttctggcaatacctgaggactg gaagaccgattaaccacggaggcctgccctgactgcccacggtgccgtggccaaagaggatctaagggcacc gctgagggcctacctaaccatcgtggggaataaggacagtgtcaccc

Supplementary Table S2: Primer sequences

**Gene**	**Forward**	**Reverse**
Mouse *Usp24*	TCAACTCCTCTGTGGTGCAG	CCAGCCTCTTGTCTTTGAGG
Mouse *Stat2*	AATCAGGCTCAAAGAGCTCAAG	TCACCAAGAACTCAATGTCCAC
Mouse *Tnfsf11* (RANKL)	CAGCATCGCTCTGTTCCTGTA	CTGCGTTTTCATGGAGTCTCA
Mouse *Tnfrsf11b* (OPG)	CGGAAACAGAGAAGCCACGCAA	CTGTCCACCAAAACACTCAGCC
Mouse *Ibsp*	CAGGGAGGCAGTGACTCTTC	AGTGTGGAAAGTGTGGCGTT
Mouse *Bglap*	GCAGCACAGGTCCTAAATAG	GGGCAATAAGGTAGTGAACAG
Mouse *Col1a1*	ACTGTCCCAACCCCCAAAG	ACGTATTCTTCCGGGCAGAA
Mouse *Runx2*	TACAAACCATACCCAGTCCCTGTTT	AGTGCTCTAACCACAGTCCATGCA
Mouse *Sp7*	ATGGCGTCCTCTCTGCTTGA	GAAGGGTGGGTAGTCATTTG
Mouse *Ctsk*	AGCAGAACGGAGGCATTGACTC	CCCTCTGCATTTAGCTGCCTTTG
Mouse *Nfatc1*	GGTGCCTTTTGCGAGCAGTATC	CGTATGGACCAGAATGTGACGG
Mouse *Acp5*	GCGACCATTGTTAGCCACATACG	CGTTGATGTCGCACAGAGGGAT
Mouse *Id1*	ACATGAACGGCTGCTACTCAC	GACTTCAGACTCCGAGTTCAGC
Mouse *Ifi204*	TTTAAGTCATTGCTGGCCAGAG	TCAGTTTGCCCAATCCAGAATC
Mouse *Cxcl10*	TGCCGTCATTTTCTGCCTCA	CAAGCTTCCCTATGGCCCTC
Mouse *Irf7*	TTCGGTCGTAGGGATCTGGA	CCTCTTCGCTCTCTTCGCTC
Mouse I*sg15*	CTGGTGAGGAACGAAAGGGG	CTCGAAGCTCAGCCAGAACT
Mouse *Rplp0*	TGGCCAATAAGGTGCCAGCTGCTG	CTTGTCTCCAGTCTTTATCAGCTGCAC
Human *USP24*	GATGGAGTTCAGCACAAGCA	GAGCAATGCTAATGGGTGGT
Human *IBSP*	CAACAGCACAGAGGCAGAAA	CAACAGCACAGAGGCAGAAA
Human *BGLAP*	AGAGTCCAGCAAAGGTGCAG	TCAGCCAACTCGTCACAGTC
Human *COL2A1*	GTCTACCCCAATCCAGCAAA	GTTGGGAGCCAGATTGTCAT
Human *SOX9*	ATCAAGACGGAGCAGCTGAG	TGGTGGTCGGTGTAGTCGTA
Human *RPLP0*	GGAATGTGGGCTTTGTGTTC	TGCCCCTGGAGATTTTAGTG

Supplementary Figure Legends

Supplementary Figure S1. USP24 silencing by rAAV suppresses osteoblast differentiation of BMSCs.

(A-C) 5 x 10^{10} genome copies (GCs) of recombinant adeno-associated virus serotype 9 (rAAV9).egfp carrying *amiR-Ctrl* or *amiR-Usp24* were treated to mouse bone marrow-derived mesenchymal stem cells (BMSCs), and cells were cultured under osteogenic conditions for 6 days. Osteoblast differentiation was assessed by alkaline phosphatase (ALP) staining (A), ALP activity (B, $n = 12$), osteoblast gene expression (C, $n = 4$), and a RANKL/OPG ratio (*Rankl/Opg* mRNA) (D, $n = 4$). Scale bar, 500 μm (A). Data are representative of three (A-D). A two-tailed unpaired Student's t-test for comparing two groups (B-D; error bars, SD of biological replicates).

Supplementary Figure S2. USP24 is required for *in vivo* bone formation.

(A-D) 10^{11} GCs of rAAV9.egfp carrying *amiR-Ctrl* or *amiR-Usp24* were injected into postnatal day 0 (P0) female pups, and 28 days later, *Usp24* mRNA levels in the tibia were assessed by qRT-PCR (A, $n = 6$). Micro-computed tomography (Micro-CT) analysis showing femoral bone mass in rAAV-treated 1-month-old female mice. Representative 3D-reconstruction (B) and relative quantification (C, $n = 7$ for *amiR-Ctrl* and $n = 6$ for *amiR-Usp24*) are displayed. (D) Hematoxylin and eosin (H&E) staining of the longitudinal sections of the kidney, lung, liver, spleen, and heart was performed to assess tissue histopathology. Trabecular bone volume/total volume (Tb. BV/TV), trabecular number (Tb. N), trabecular thickness (Tb. Th), and cortical thickness (C. Th) were measured. Scale bar, 500 μm (B) or 50 μm (D). Data are representative

of three (B, D). A two-tailed unpaired Student's t-test for comparing two groups (A, C; error bars, SD of biological replicates).

Supplementary Figure S3. USP24 is essential for maintaining skeletal homeostasis.

(A-C) 5 x 10^{13} vg/kg of rAAV9.egfp carrying *amiR-Ctrl* or *amiR-Usp24* was injected into 3-month-old male mice, and 2 months later, femoral longitudinal sections were assessed by H&E staining (A, top) and TRAP staining (A, bottom). (B) mRNA levels of a RANKL/OPG ratio in the tibia were assessed by qRT-PCR (n = 4). (C) H&E staining of the longitudinal sections of the kidney, lung, liver, spleen, and heart was performed to assess tissue histopathology. Scale bar, 50 μm (A, C). Data are representative of three (A-C). A two-tailed unpaired Student's t-test for comparing two groups (B; error bars, SD of biological replicates).

Supplementary Figure S4. USP24 promotes STAT2 activation by regulating STAT2 protein stability.

(A) *shCtrl* or *shUsp24*-expressing mouse calvarial osteoblasts (COBs) were treated with interferon alpha-2 (20 ng/ml, 4 hours), and phosphorylated STAT2 and total protein levels of STAT2 were examined by immunoblotting.

(B) *shCtrl*- or *shStat2*-expressing C3H10T1/2 cells were transfected with the ISRE reporter gene along with *Renilla*. After 48 hours, luciferase activity was measured and normalized to *Renilla* (n = 4).

(C) mRNA levels of ISGs in *shCtrl* or *shUsp24*-expressing mouse COBs (n = 4). Data are representative of three (A-C). A two-tailed unpaired Student's t-test for comparing two groups (B, C; error bars, SD of biological replicates).

Supplementary Figure S5. STAT2 promotes osteoblast differentiation.

(A) *shCtrl* or *shStat2*-expressing mouse COBs were cultured under osteogenic conditions for 6 days. Osteoblast gene expression (A, n = 4), and a RANKL/OPG ratio (*Rankl/Opg* mRNA) (B, n = 4). Data are representative of three (A, B). A two-tailed unpaired Student's t-test for comparing two groups (A, B; error bars, SD of biological replicates).

Supplementary Figure S6. STAT2 regulates the expression of osteoclast markers yet is dispensable for osteoclast maturation and function.

(A-C) 5×10^{10} GCs of rAAV9.egfp carrying *amiR-Ctrl* or *amiR-Stat2* were introduced to mouse BMMs, and cells were cultured under osteoclast differentiation conditions for 4 days. Osteoclast differentiation was assessed by tartrate-resistant acid phosphatase (TRAP) activity (A, n = 9), TRAP staining (B), and osteoclast gene expression (C, n = 4). Scale bar, 100 μm (B, upper) or 50 μm (B, lower). Data are representative of three (A-C). A two-tailed unpaired Student's t-test for comparing two groups (A, C; error bars, SD of biological replicates).

Supplementary Figure S7. STAT2 regulates RUNX2 in osteoblasts.

(A) mRNA levels of *Stat2* and *Ifi204* in *shCtrl*- or *shStat2*-expressing mouse COBs (n = 4).

(B) *shCtrl*- or *shStat2*-expressing C3H10T1/2 cells were transfected with the OG2 reporter gene along with *Renilla*. After 48 hours, luciferase activity was measured and normalized to *Renilla* ($n = 4$).

(C) Wild-type mouse COBs were lysed, immunoprecipitated with anti-IgG control or anti-RUNX2 antibody, and protein G-conjugated agarose and immunoblotted with the indicated antibodies. Data are representative of three (A-C). A two-tailed unpaired Student's t-test for comparing two groups (A, B; error bars, SD of biological replicates).

Supplementary Figure S8. STAT2 silencing by rAAV suppresses expression of ISGs.

5 x 10^{10} genome copies (GCs) of rAAV9.egfp carrying *amiR-Ctrl* or *amiR-Stat2* were treated to mouse BMSCs, and cells were cultured under osteogenic conditions. mRNA levels of ISGs (A: *Cxcl10*, *Irf7*, *Isg15*, and *Ifi204*) were examined at day 6 ($n = 4$). Data are representative of three. A two-tailed unpaired Student's t-test for comparing two groups (error bars, SD of biological replicates).

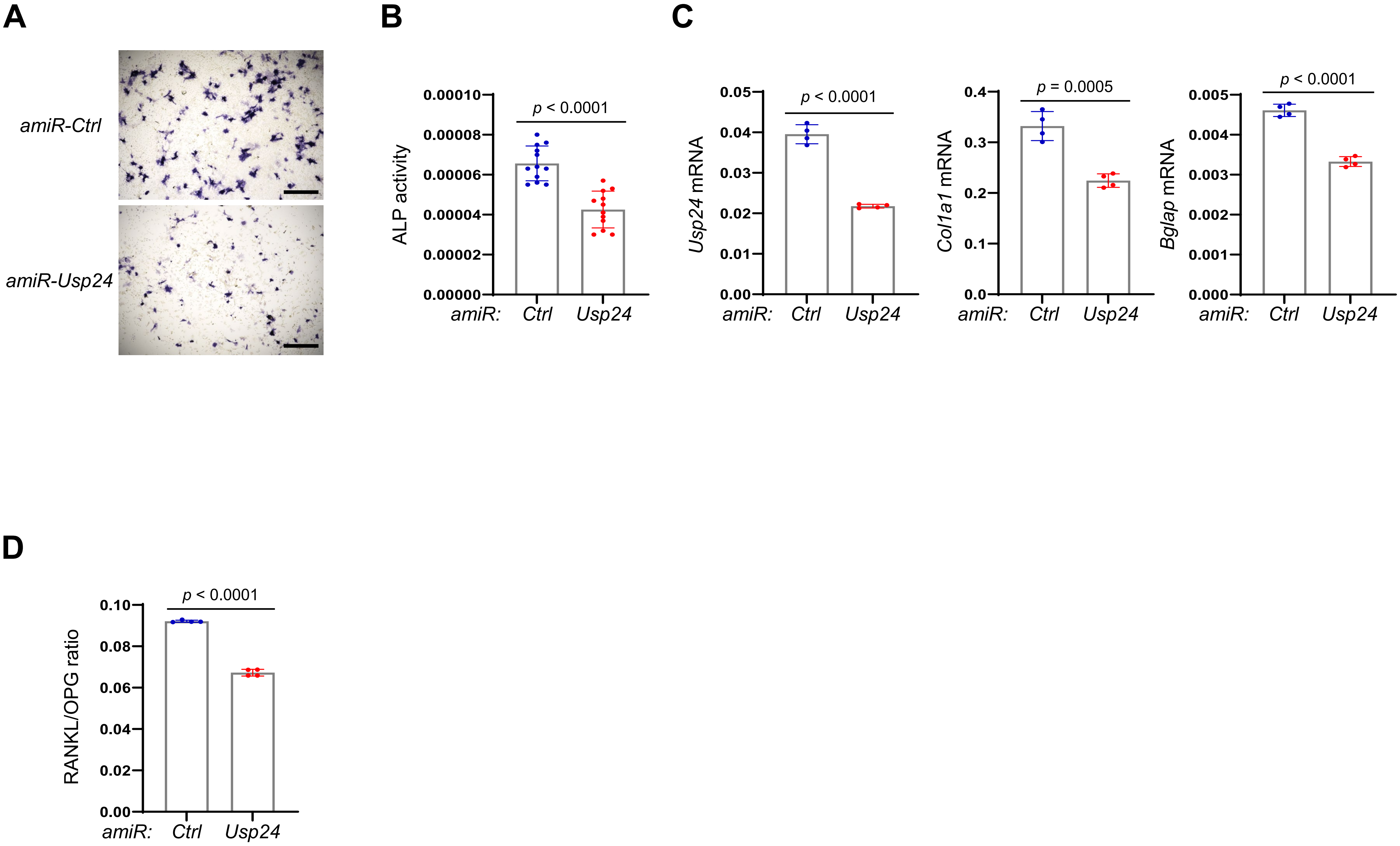

Supplementary Fig. S1

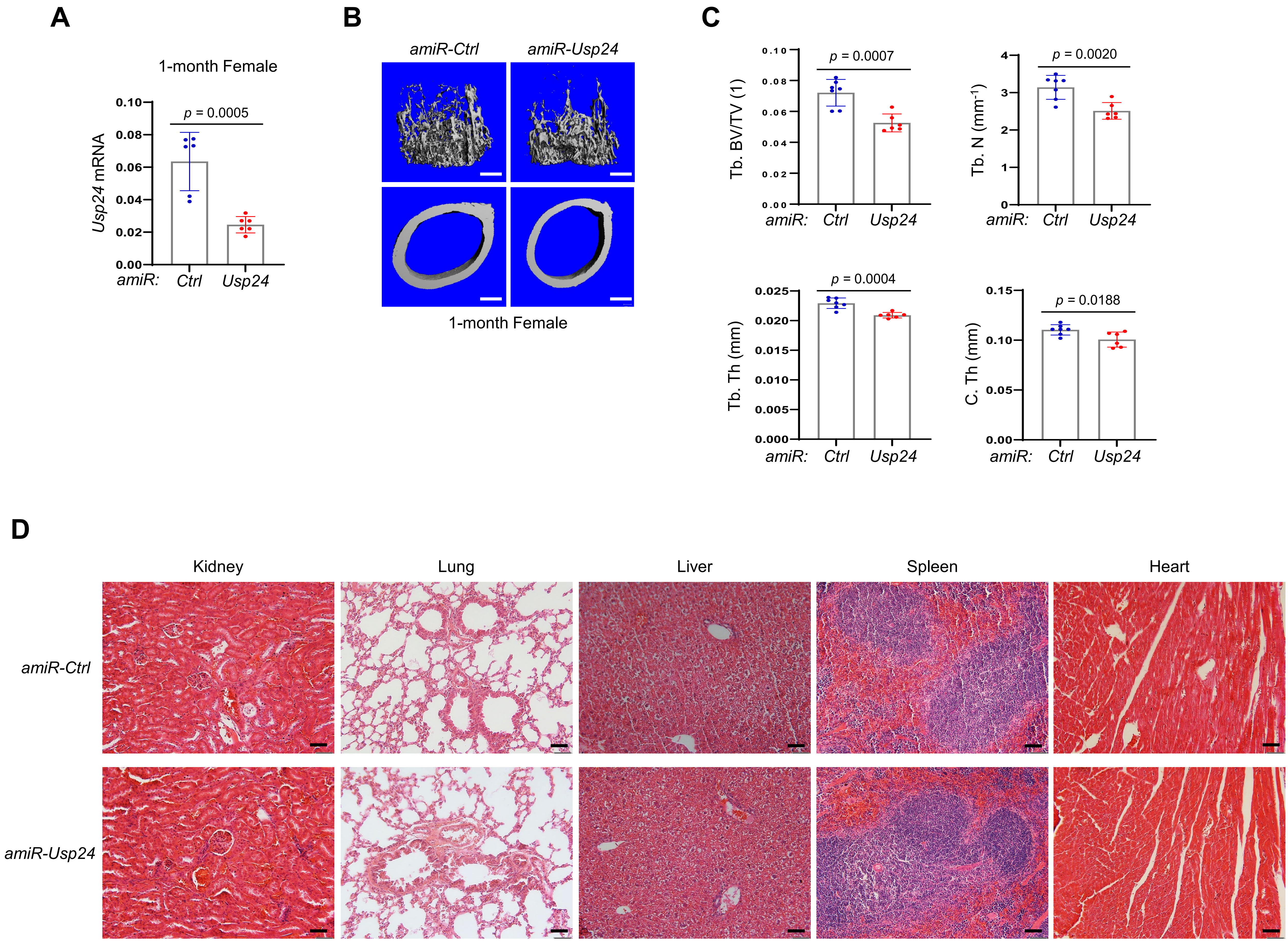

Supplementary Fig. S2

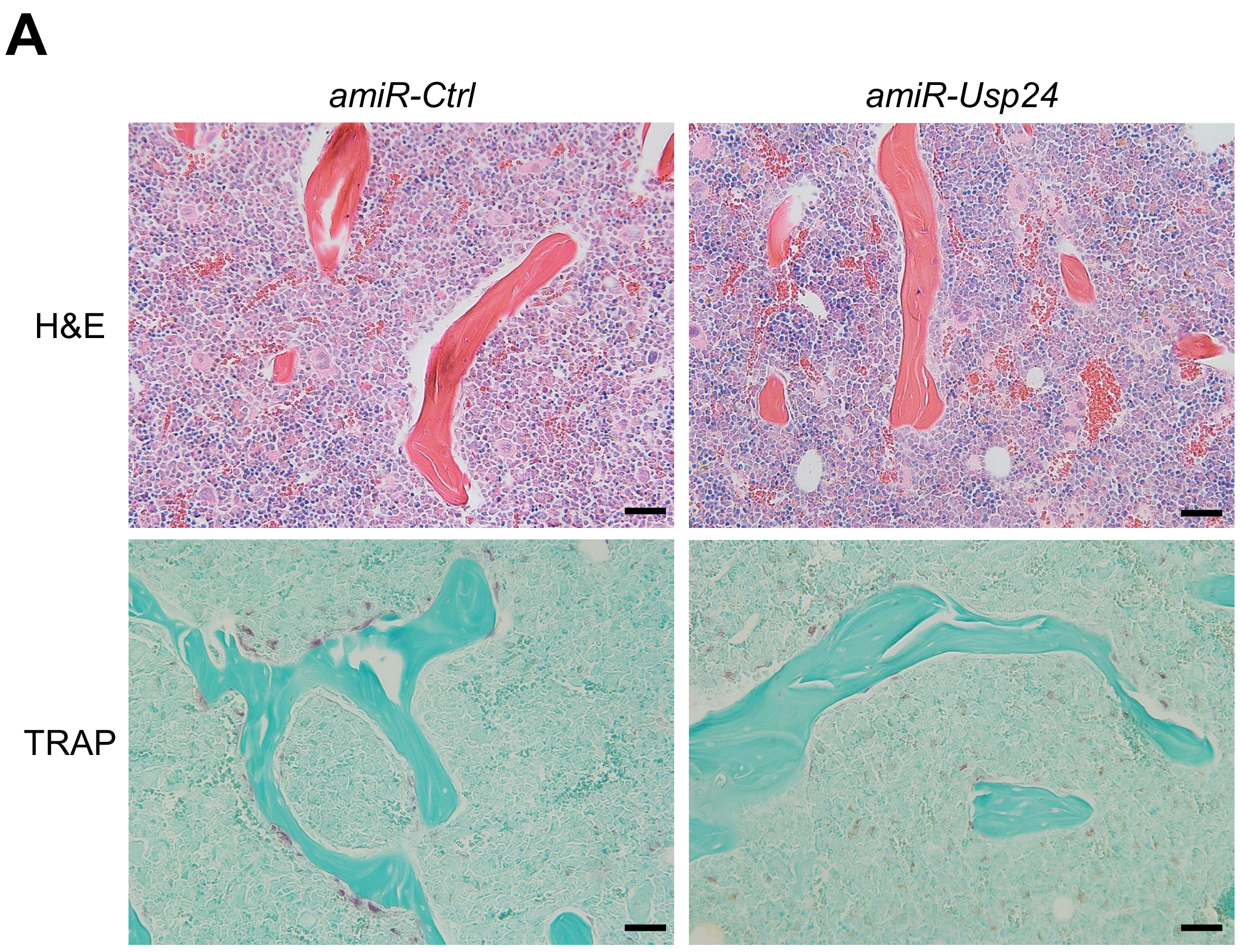

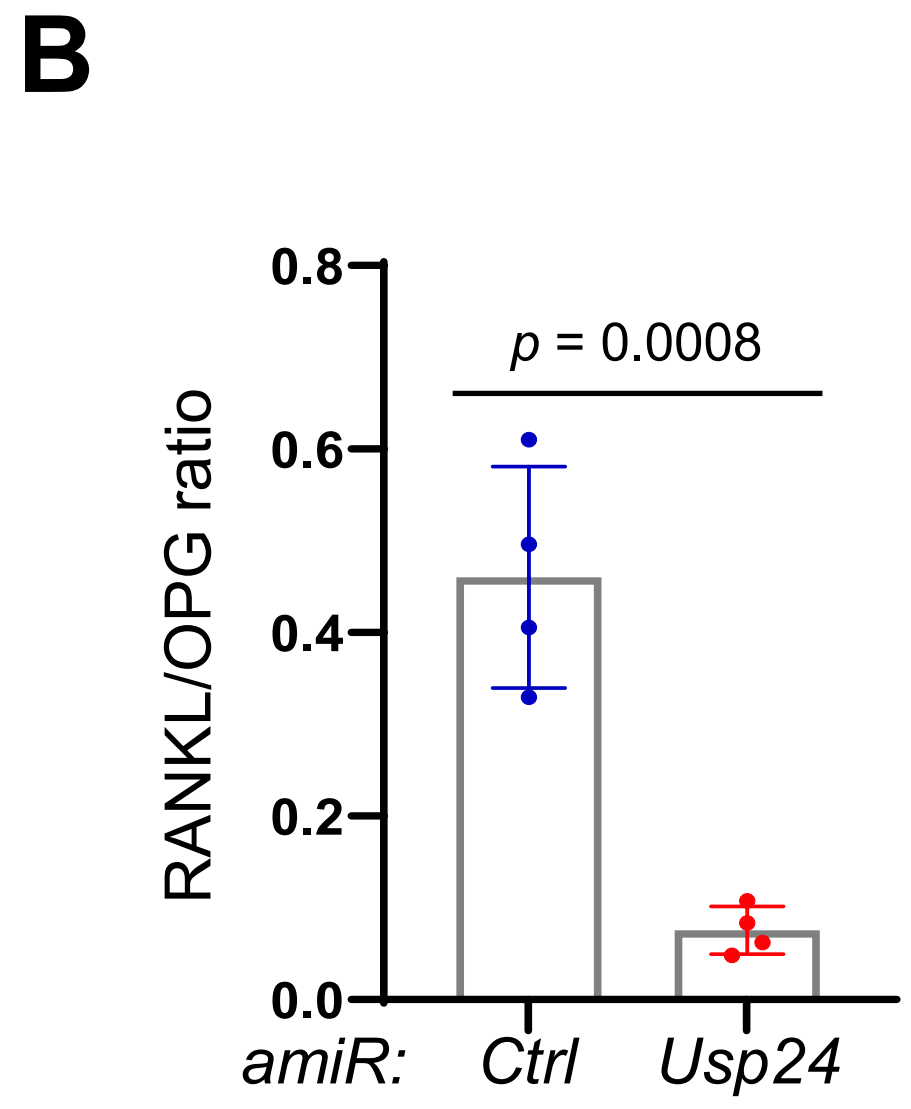

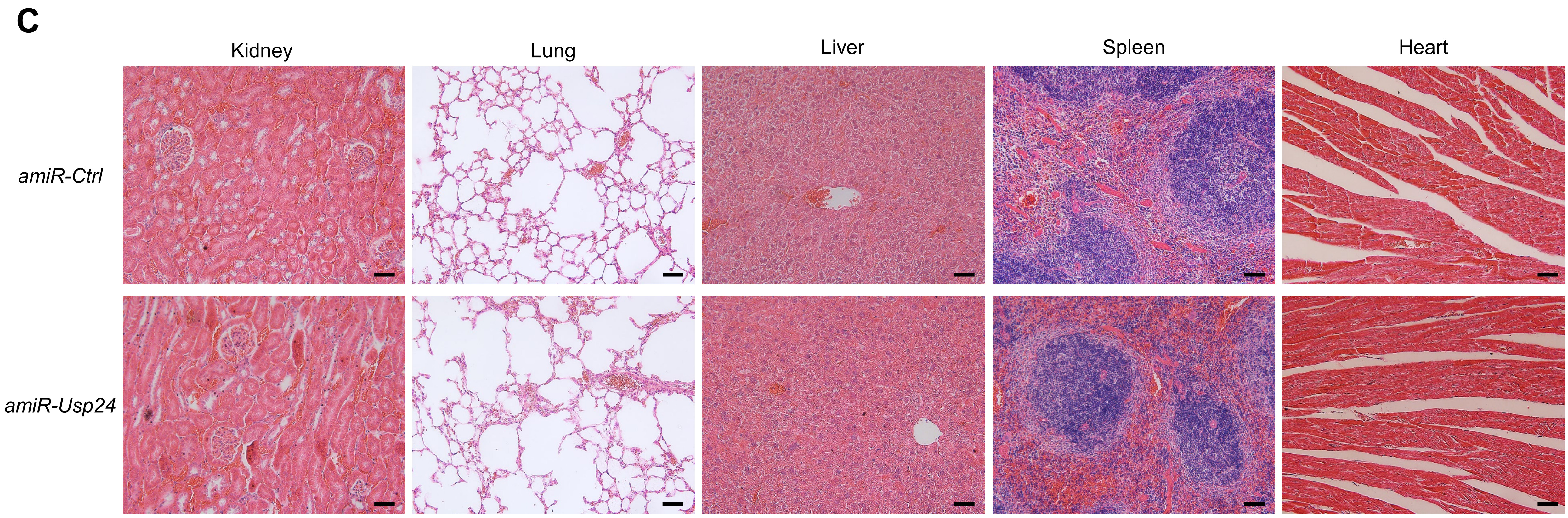

Supplementary Fig. S3

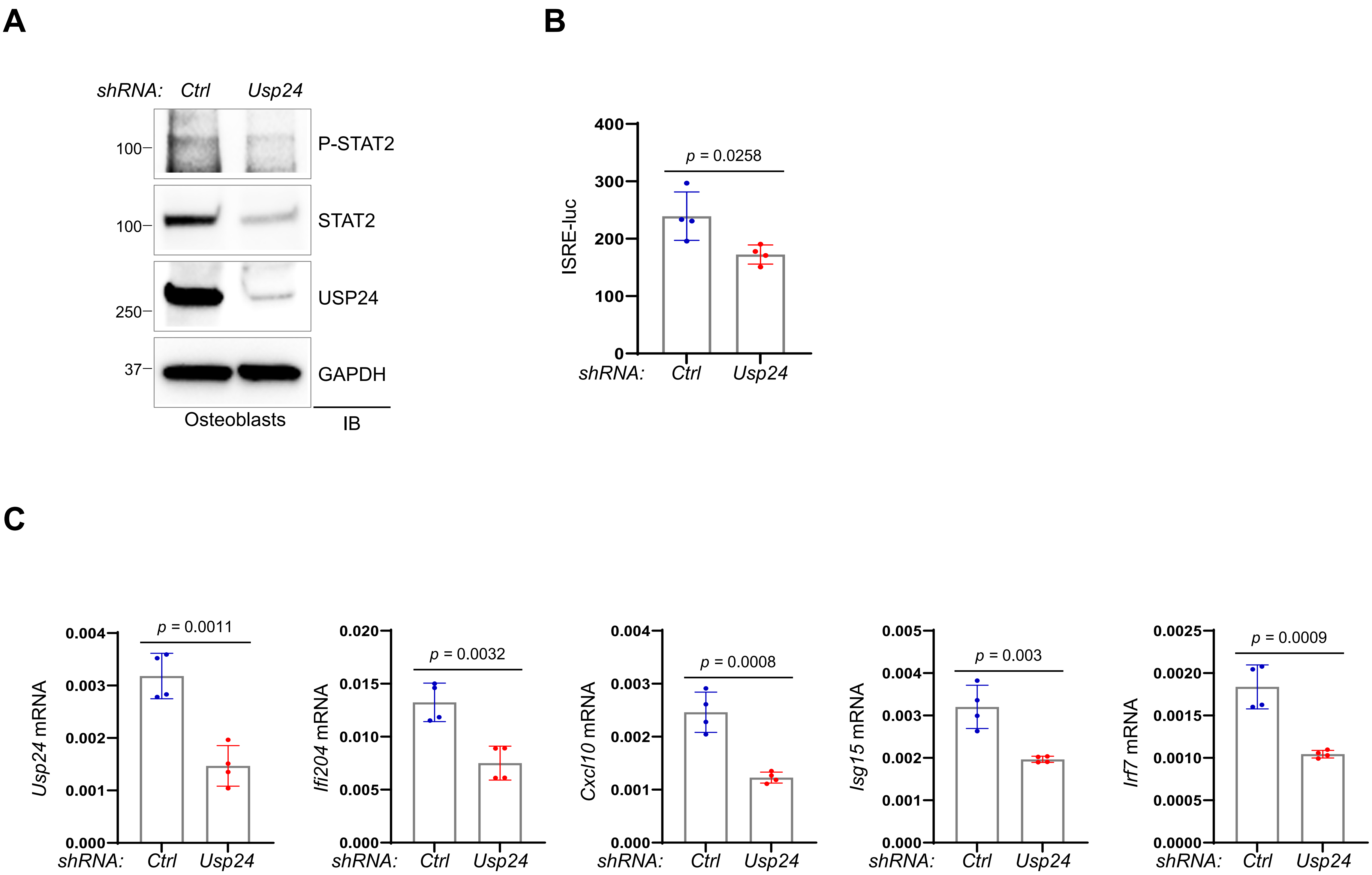

Supplementary Fig. S4

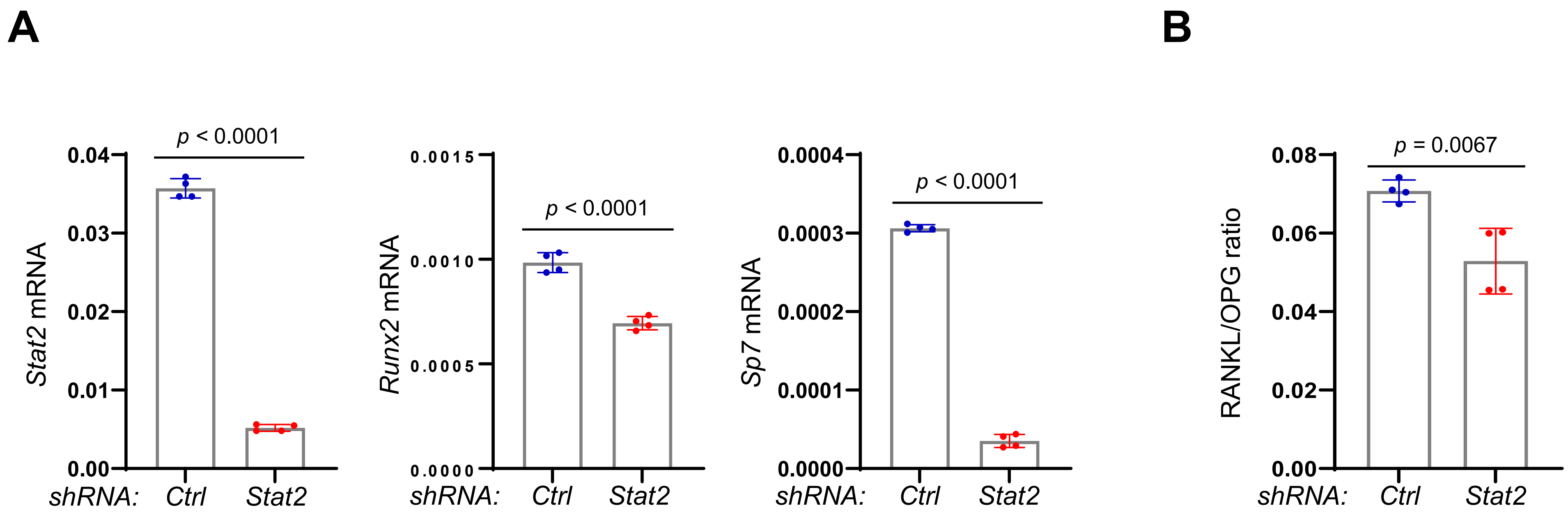

Supplementary Fig. S5

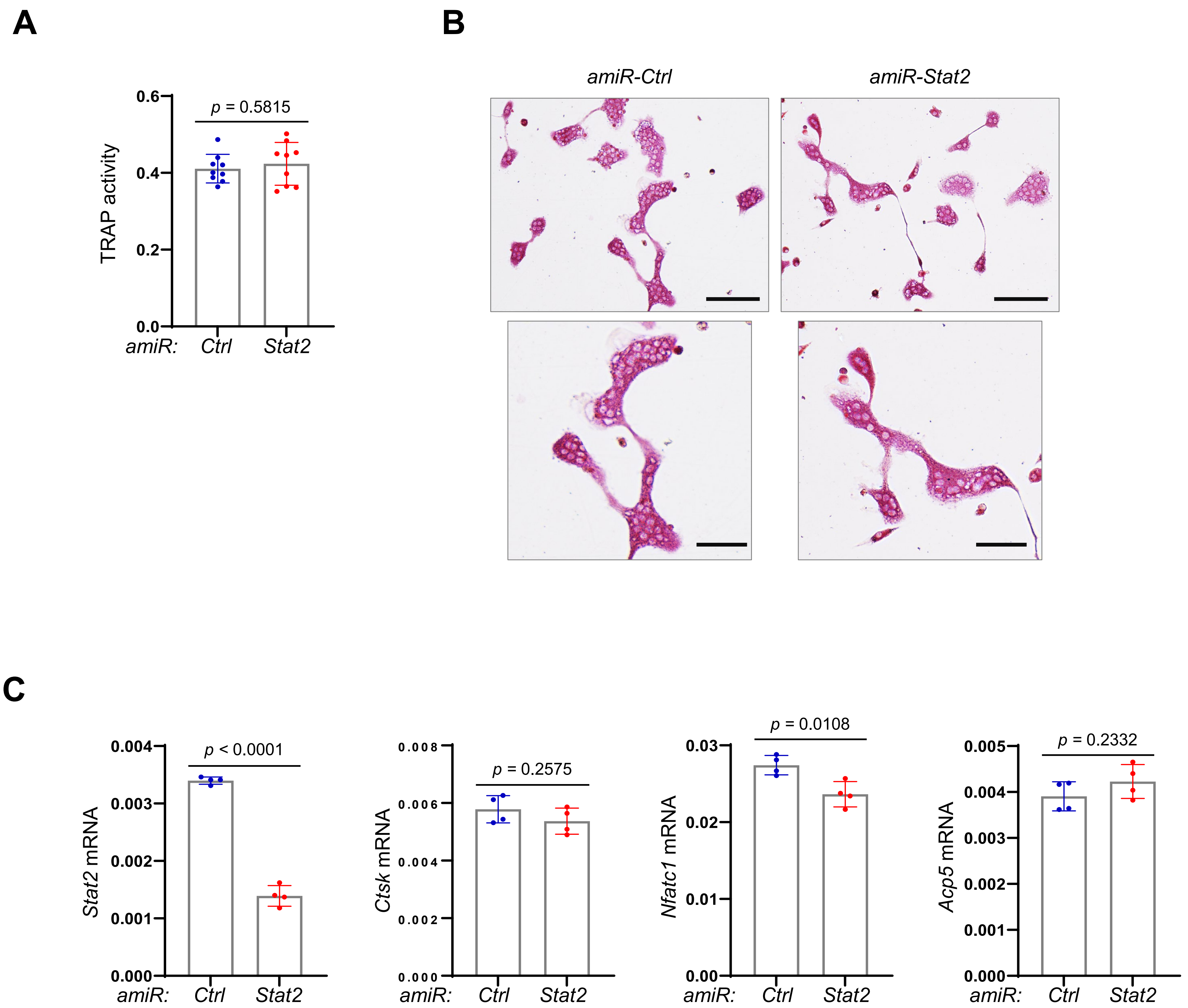

Supplementary Fig. S6

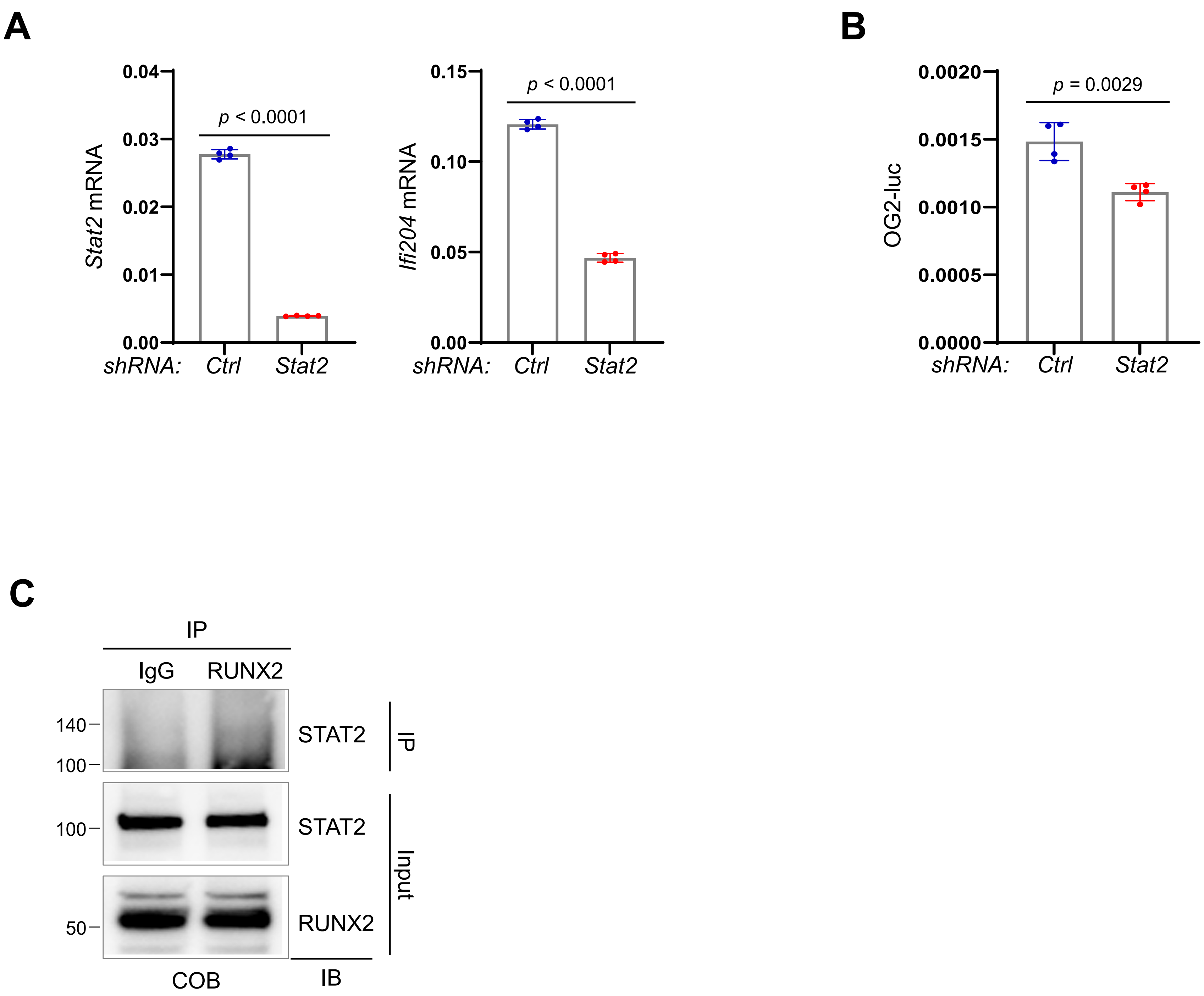

Supplementary Fig. S7

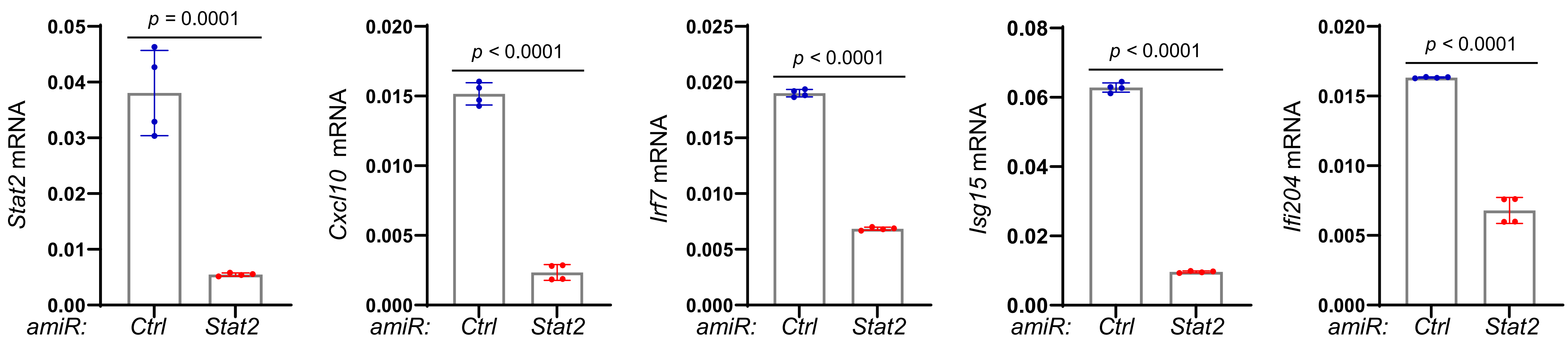

Supplementary Fig. S8
