# Supplementary material for: USP24-dependent STAT2 stabilization mediates physiologic and pathologic bone formation
Source: Cell Death Dis. 2026 May 30;17(1):675. doi: 10.1038/s41419-026-08888-7 (PMC13433775; doi:10.1038/s41419-026-08888-7)
Supplement: Supplementary file 2 [file 41419_2026_8888_MOESM2_ESM.pdf]

Kim et al.,

USP24-dependent STAT2 stabilization mediates physiologic and pathologic bone formation

- Uncropped immunoblot images

Fig. 3A

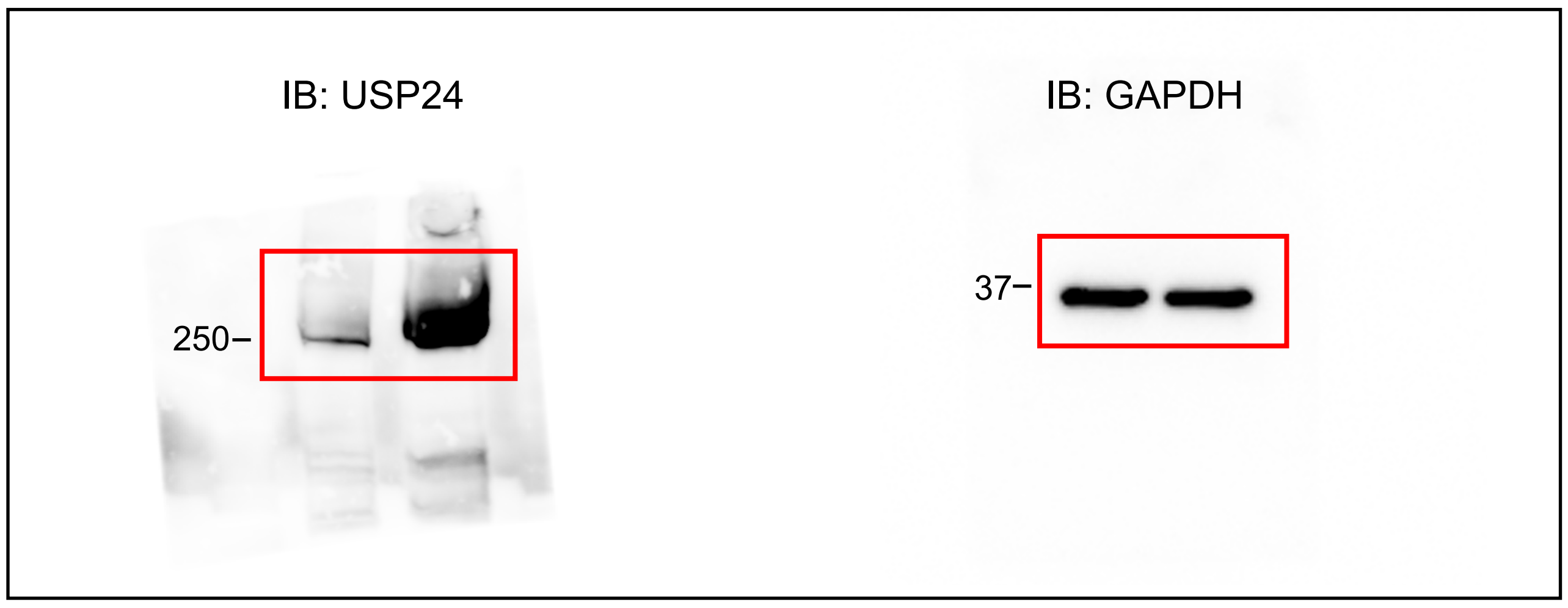

Fig. 3C

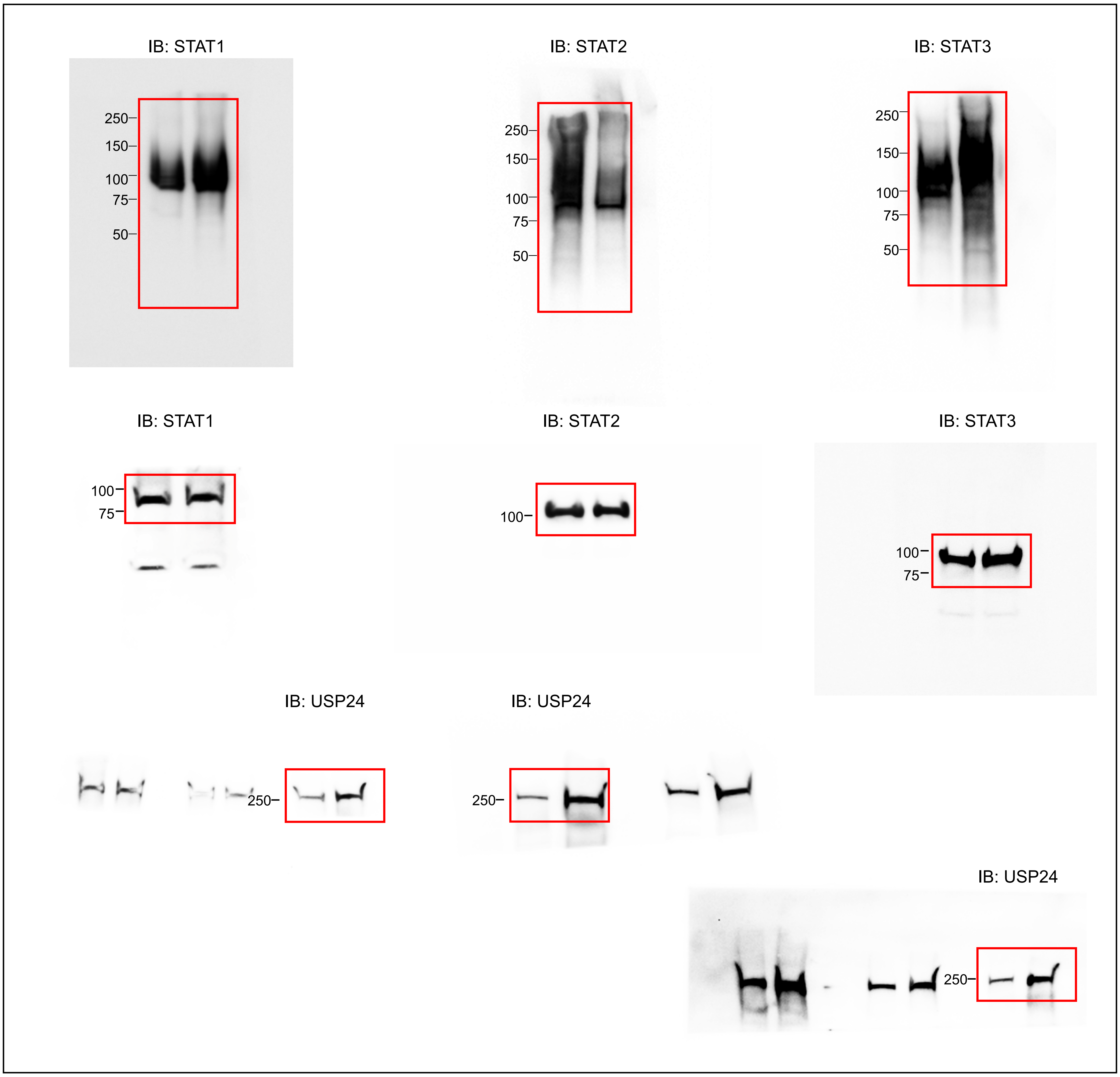

Uncropped immunoblots

Fig. 3D

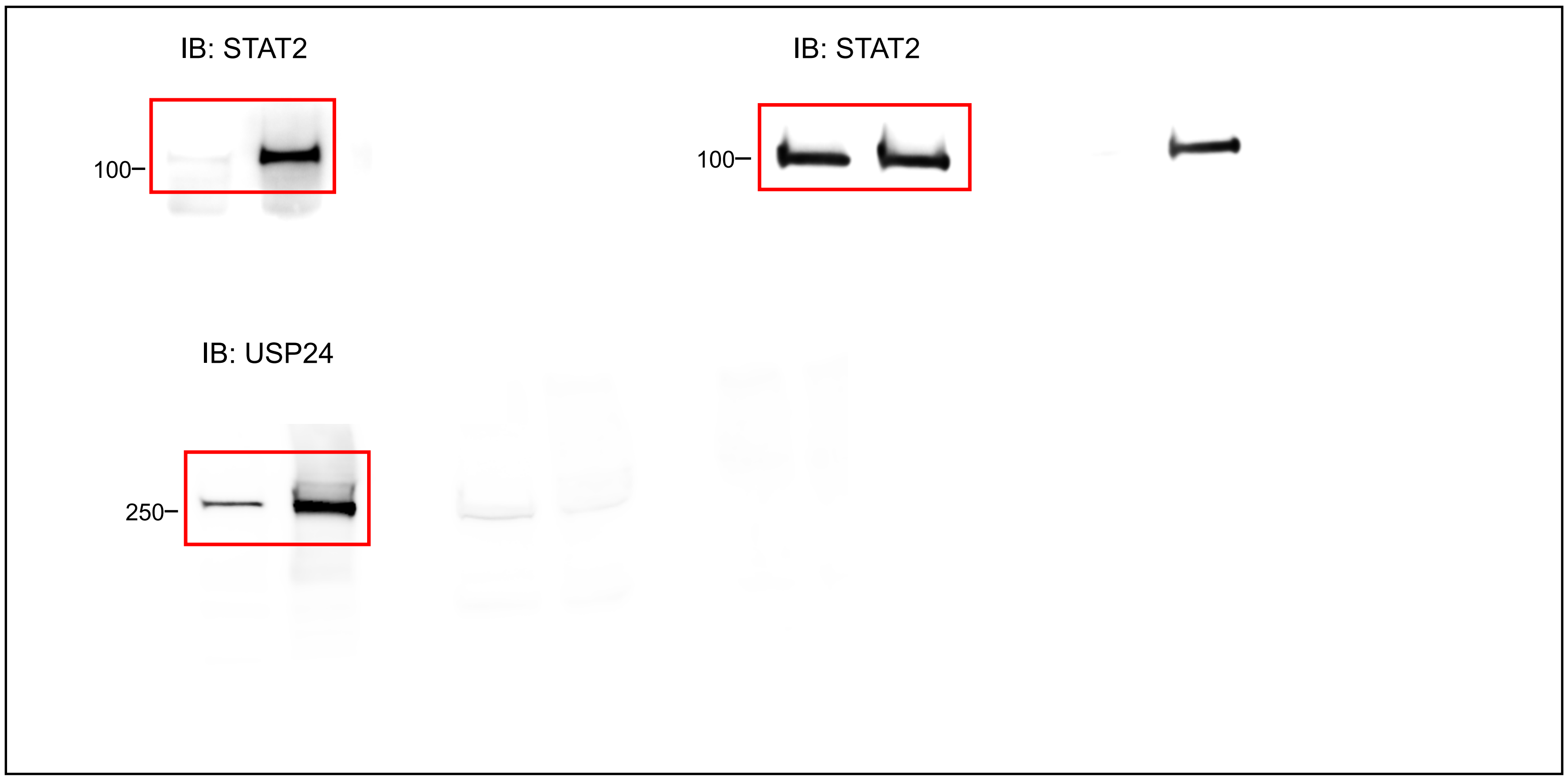

Fig. 3E

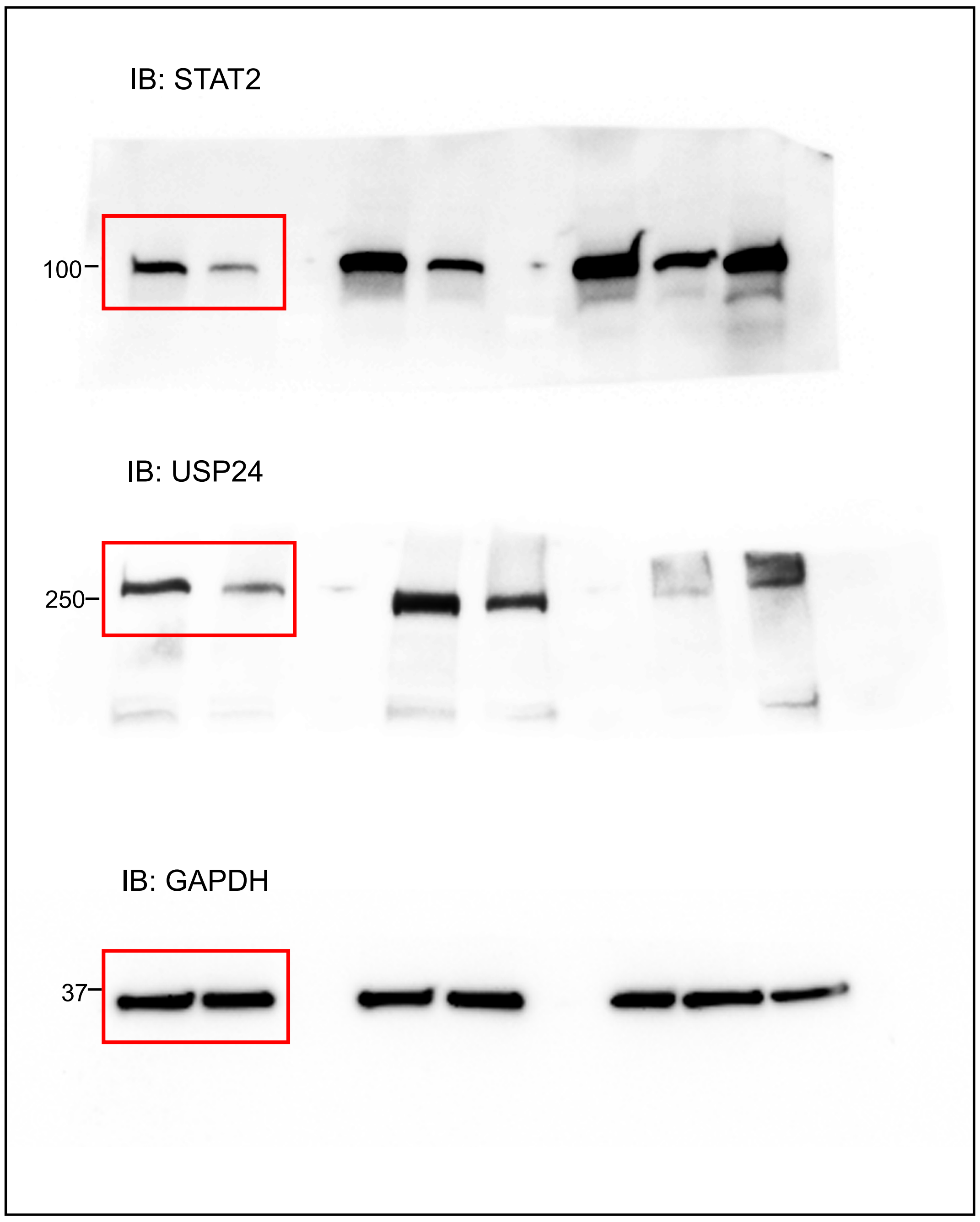

Fig. 3G

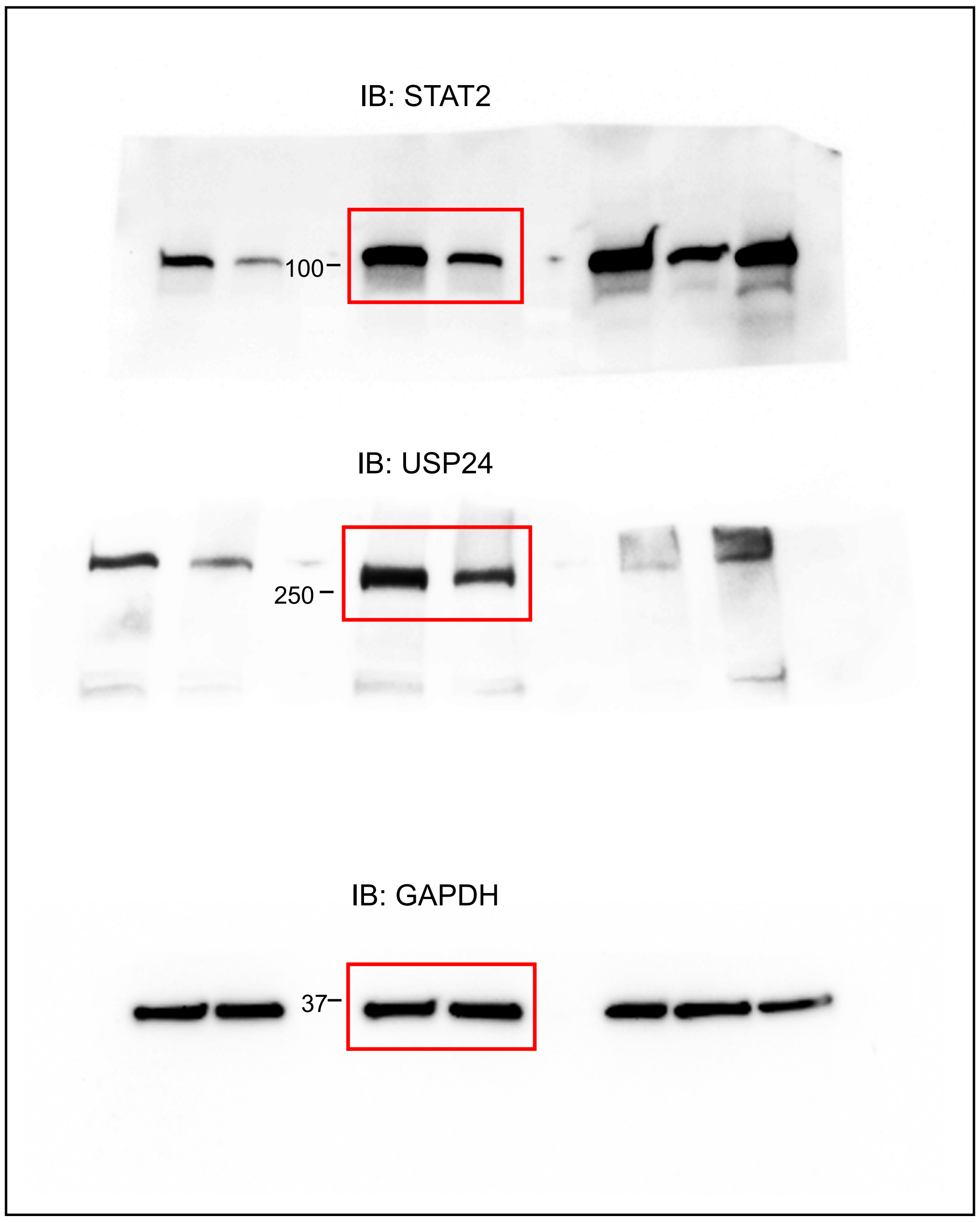

Uncropped immunoblots

Fig. 3H

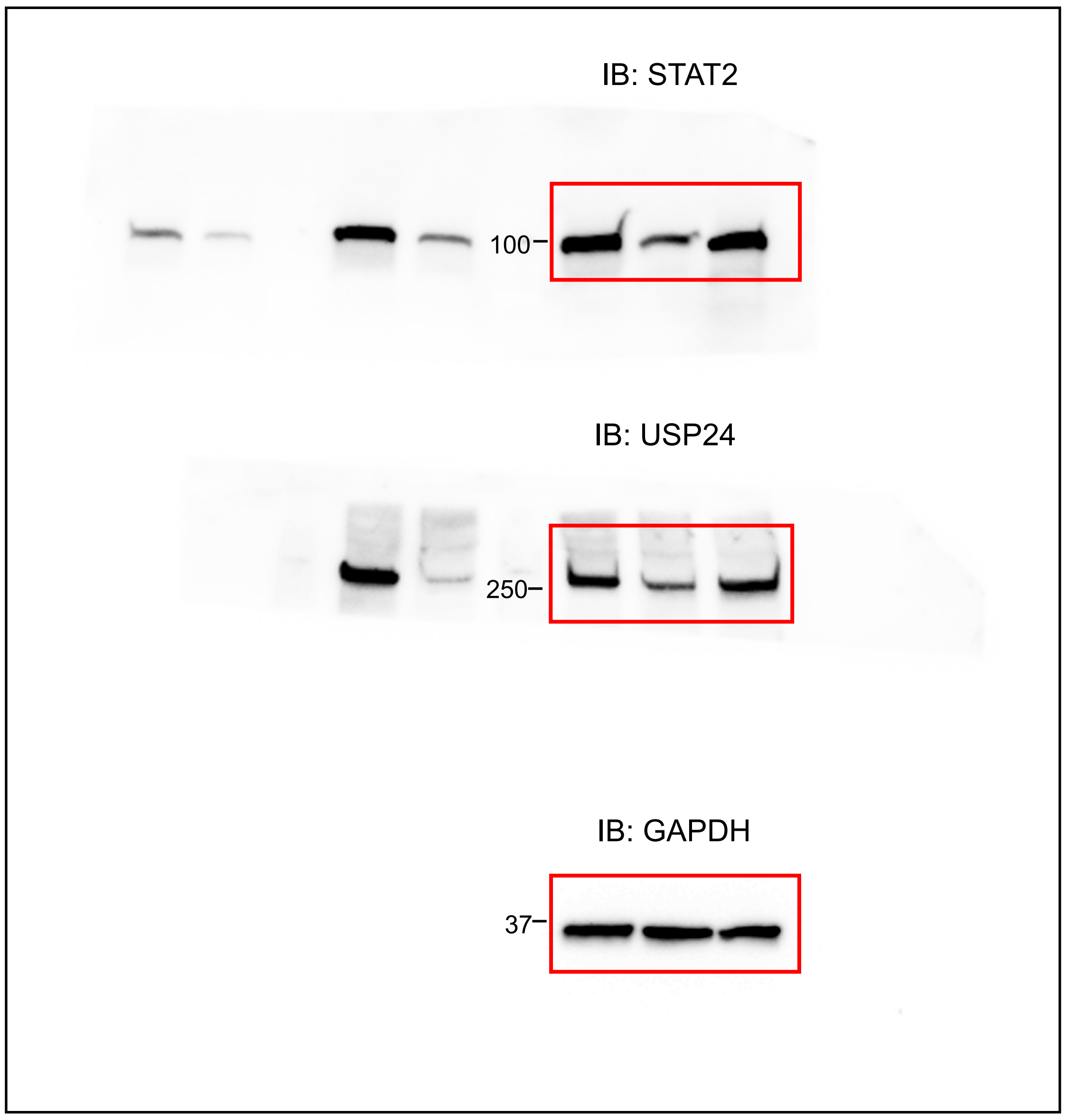

Fig. 5F

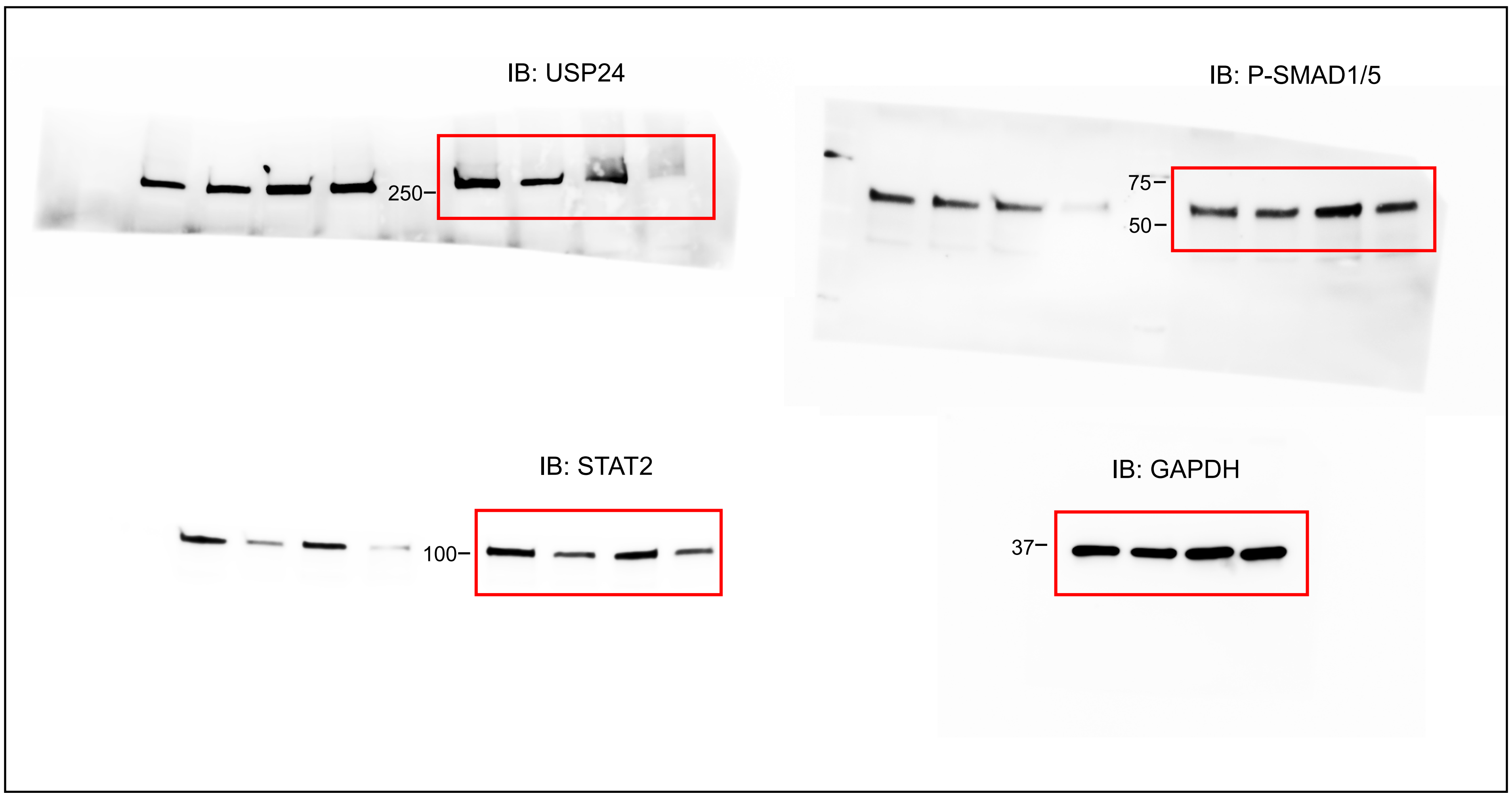

Uncropped immunoblots

Supplementary Fig. S4A

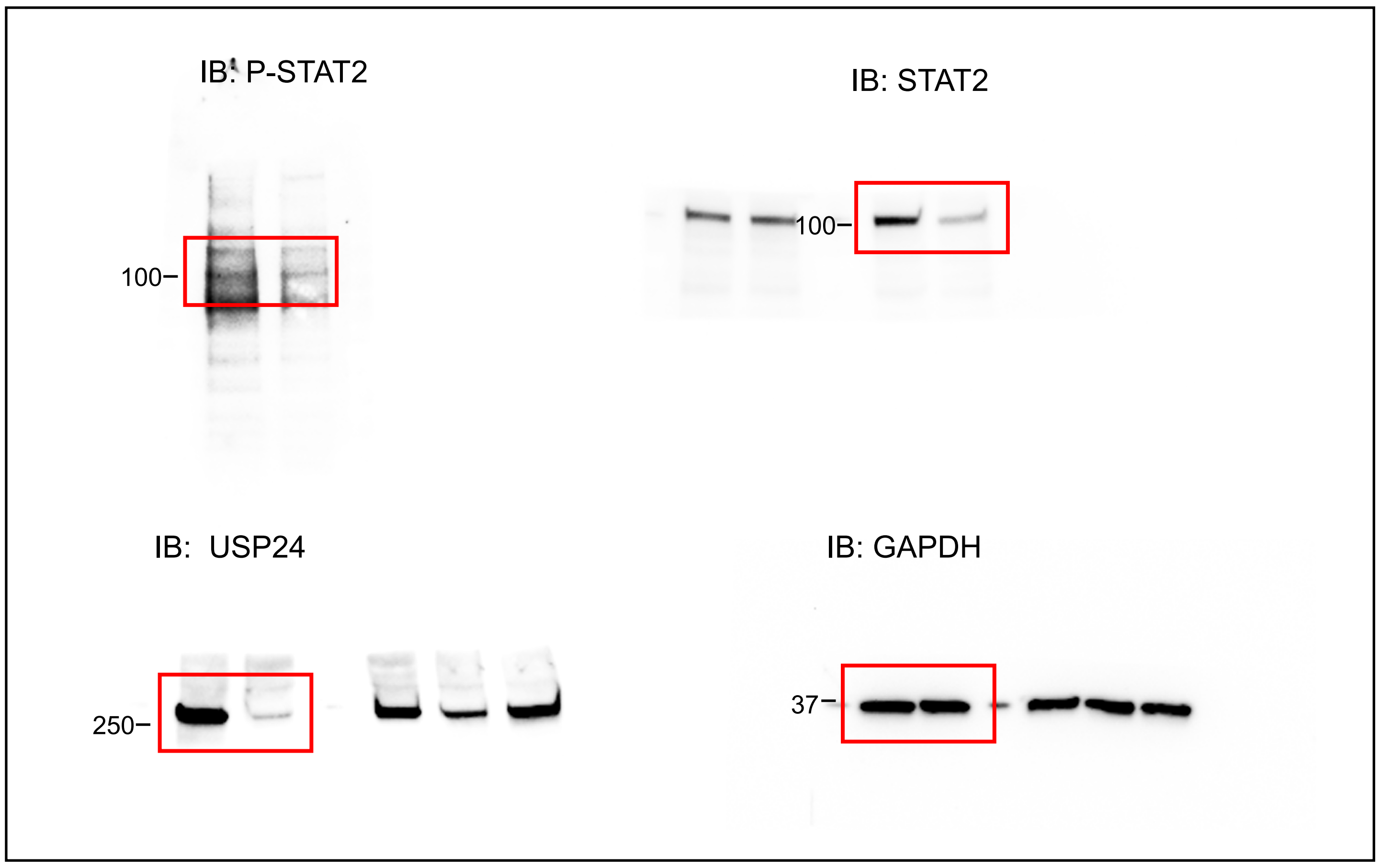

Supplementary Fig. S7C

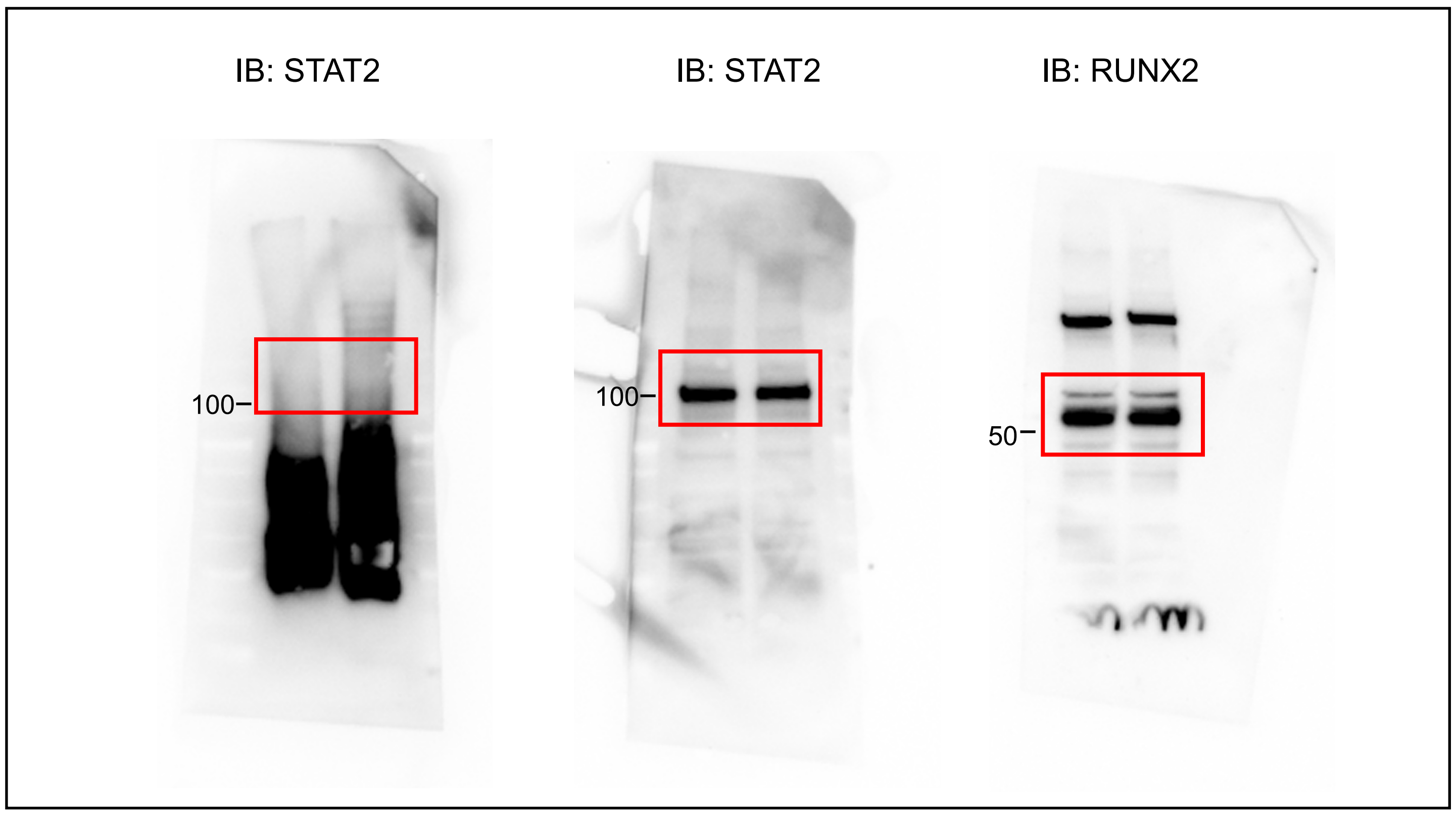

Uncropped immunoblots
